# An Ultra-Brief Informant Questionnaire for Case Finding of Cognitive Impairment Across Diverse Literacy: Diagnostic Accuracy Study

**DOI:** 10.2196/72963

**Published:** 2026-01-06

**Authors:** Tau Ming Liew, King Fan Yip, Kaavya Narasimhalu, Simon Kang Seng Ting, Weishan Li, Sze Yan Tay, Way Inn Koay

**Affiliations:** 1 Department of Psychiatry, Singapore General Hospital, Outram Road, Singapore, 169608, Singapore, 65 62223322; 2 SingHealth Duke-NUS Medicine Academic Clinical Programme, Duke-NUS Medical School, Singapore, Singapore; 3 Health Services and Systems Research, Duke-NUS Medical School, Singapore, Singapore; 4 Saw Swee Hock School of Public Health, National University of Singapore, Singapore, Singapore; 5 Department of Geriatric Medicine, Singapore General Hospital, Singapore, Singapore; 6 Department of Neurology, National Neuroscience Institute, Singapore General Hospital, Singapore, Singapore; 7 Department of Psychology, Singapore General Hospital, Singapore, Singapore

**Keywords:** machine learning, Informant Questionnaire for cognitive impairment–two items plus demographics, IQ2+, informant questionnaire, subjective questionnaire, subjective cognitive decline, neurocognitive disorders

## Abstract

**Background:**

Undiagnosed cognitive impairment poses a global challenge, prompting recent interest in ultra-brief screening questionnaires (comprising <2 to 3 items) to efficiently identify individuals needing further evaluation. However, evidence on ultra-brief questionnaires remains limited, particularly regarding their validity across diverse literacy levels.

**Objective:**

This study aimed to develop an ultra-brief questionnaire that performs well in detecting mild cognitive impairment or dementia (MCI/dementia) across diverse literacy levels and to compare its performance with an established questionnaire (the 8-item Informant Interview to Differentiate Aging and Dementia [AD8]).

**Methods:**

This diagnostic study involved 1856 participants aged ≥65 years (median education 10 y, range 0‐23 y), prospectively recruited from community settings in Singapore. Participants and informants completed 15 cognition-related questions. MCI/dementia was diagnosed via a comprehensive assessment and consensus conference. The sample was randomly split 70/30—the training sample (70%) was used to derive an ultra-brief questionnaire from the 15 cognition-related questions (using an exhaustive search approach), and the test sample (30%) evaluated the area under the receiver operating characteristic curve (AUC).

**Results:**

The new questionnaire comprised 2 informant questions (ie, *assistance with medications* and *worry about cognition*), plus age and years of education. It demonstrated excellent performance in detecting MCI/dementia (AUC 85%, 95% CI 80%‐90%), significantly better (*P*=.003) than a nested baseline model (comprising age and years of education; AUC 78%, 95% CI 73%‐83%). In contrast, the AD8 had an AUC of 76% (95% CI 70%‐83%), not significantly different (*P>*.99) from the baseline model. The questionnaire’s performance was consistent across education subgroups and varying prevalence scenarios. Two optimal cutoffs were used—the lower cutoff provided 80% sensitivity and 96% negative predictive value, and the upper cutoff provided 99% specificity and 81% positive predictive value. A web-based calculator is available for public use.

**Conclusions:**

This ultra-brief questionnaire enables rapid screening for cognitive impairment (in <1 min) by family members or as part of community geriatric assessments. Its excellent performance across literacy levels supports its utility for case finding in diverse populations, including underserved communities and lower- and middle-income countries.

## Introduction

Undiagnosed cognitive impairment is a global challenge [1], with 60% to 90% of affected individuals never receiving a formal diagnosis [2,3]. Those who remain undiagnosed miss out on timely clinical care [4], including management of reversible causes, prescription of cognitive enhancers, behavioral management, caregiver support, and advanced care planning [4-9]. All these can impact well-being [10,11] and increase the risk of premature nursing home placement [12-14]. Furthermore, undiagnosed individuals often do not receive adequate support to manage and coordinate care for their chronic diseases [15,16], resulting in suboptimal disease management, inappropriate health care utilization, and higher health care costs [17,18]. As an example, a modeling study estimated that timely management of cognitive impairment could potentially yield annual cost savings of US $13 to $41 billion in the United States [15]. Recently, the importance of early diagnosis has been heightened by emerging evidence supporting early interventions for cognitive impairment [19,20], such as risk factor modification [21,22] and antiamyloid monoclonal antibodies [23,24].

To address the challenge of undiagnosed cognitive impairment, various international bodies (eg, the Alzheimer’s Disease International [25] and the International Association of Gerontology and Geriatrics) [26] have advocated for a systematic approach to case finding among high-risk individuals in the community [26]. In particular, a 2-stage strategy [27-29] has been proposed in recent literature to address resource constraints for community case finding. In the first stage, subjective reports (eg, Functional Activities Questionnaire and AD8 [the 8-item Informant Interview to Differentiate Aging and Dementia]) [30,31] are used to identify individuals with potential cognitive impairment. In the second stage, these individuals undergo brief cognitive tests (eg, Mini-Cog and short variants of Montreal Cognitive Assessment) [4,32-34] to confirm the presence of cognitive impairment. This 2-stage strategy is efficient and scalable—the first stage relies solely on subjective reports and does not draw on scarce health care resources for administration of cognitive tests, whereas the second stage reserves brief cognitive tests for a smaller subset of individuals [27]. Moreover, combining subjective reports and brief cognitive tests has been shown to improve the detection of subtle cognitive changes [27], making this approach optimal for case finding of early cognitive impairment.

In 2019, the 2-stage strategy was adopted by the World Health Organization (WHO) within the Integrated Care for Older People assessment tool to identify cognitive impairment, alongside assessments of other key components of intrinsic capacity (ie, mobility, nutrition, hearing, vision, and mood) [29]. For the first stage (subjective report), the WHO adopted an ultra-brief questionnaire based on a single question: “Do you have problems with memory or orientation (such as not knowing where one is or what day it is)?” [29] The decision to embed an ultra-brief cognitive questionnaire within Integrated Care for Older People is understandable, as it balances the need to assess a wide range of intrinsic capacity domains against the scarcity of community resources for comprehensive assessments. However, the validity of such ultra-brief questionnaires (ie, those comprising fewer than 2‐3 items) remains unclear in the literature [28], especially when used across diverse levels of literacy [26,35]. This concern can be critical, as questionnaires with fewer items tend to have increased measurement variability and may be more susceptible to confounding factors such as educational attainment [36].

In this study, we sought to strengthen the evidence base supporting the use of ultra-brief questionnaires across diverse levels of literacy, potentially enhancing their utility in diverse populations across lower-, middle-, and higher-income countries. Specifically, we aimed to: (1) derive an ultra-brief questionnaire with high performance for detecting cognitive impairment (ie, the presence of mild cognitive impairment or dementia [MCI/dementia]) across diverse levels of literacy, using a contemporary, computationally intensive approach to identify the questions most discriminative of MCI/dementia; and (2) compare the performance of the new ultra-brief questionnaire to the well-established AD8 across participants with lower and higher educational attainment. We selected the AD8 as the benchmark because it is a widely used and extensively validated informant-based questionnaire, as demonstrated in recent systematic reviews [37,38]. Its use has been recommended by various international bodies, including the International Association of Gerontology and Geriatrics [26], the Gerontological Society of America [39], the US Alzheimer’s Association [40,41], and the National Institute on Aging workgroup [42].

Of note, this study was conducted in Singapore, a city-state in Southeast Asia that provides a unique testbed of literacy diversity for developing the ultra-brief questionnaire. The current generation of older Singaporeans witnessed the country’s transformation from a traditional, lower-income, Asian society to a more westernized, higher-income country [43]. Consequently, this cohort encompasses a wide range of educational backgrounds, from minimal formal education to tertiary education. By validating the ultra-brief questionnaire in such a heterogeneous population, we sought to demonstrate its potential for broader implementation in other literacy-diverse settings beyond Singapore, including populations across East and South Asia, and potentially, in some lower- and middle-income countries (LMICs).

## Methods

### Study Procedures

This study involved community-dwelling older persons recruited between March 2022 and September 2024, as part of a nationally funded project in Singapore aimed at developing artificial intelligence tools to detect early cognitive impairment in the community (Project PENSIEVE) [44]. Community-dwelling individuals were invited to participate if they met the following criteria: (1) higher risk of cognitive impairment (ie, aged ≥65 y [26]) and had at least one of the 3 chronic diseases (ie, diabetes mellitus, hypertension, or hyperlipidemia); this criterion was included a priori to focus on individuals with at least some risk of cognitive impairment, in line with current literature suggesting the limited benefit of screening among low-risk individuals) [26,45]; (2) ability to follow simple instructions in English or Mandarin (due to limitations in the available assessment language); and (3) presence of an informant (eg, family member or friend) who knew the participants well. Individuals were excluded if they had severe visual impairment that would affect their ability to complete neuropsychological assessments (to ensure generalizability, participants were included as long as they could see pictures on a piece of paper held in front of them). No participants were excluded for reasons related to missing data, as we implemented strict data collection procedures (eg, mandatory data field) and routine data audits throughout the study.

Sources of recruitment included 14 community roadshows conducted by the study team, clients of our community partners, home visits by community volunteers who partnered with us, media publicity (radio, online articles, and posters), and word-of-mouth referrals from participants who had completed research assessments. To ensure the recruited samples were representative of the community, the study’s publicity materials emphasized the key message of “detect dementia early” (along with direct referrals to memory clinics in the event of significant findings), rather than the conventional invitation to participate in research (which may inadvertently attract a distinct group of individuals). Examples of these publicity materials (eg, study banner, poster, and brochure) are provided in Method S1 in Multimedia Appendix 1.

All participants received comprehensive assessments, which included semistructured interviews with both participants and their informants, detailed neuropsychological testing, and observational notes of participants’ behavior during assessments. Full descriptions of the comprehensive assessments are available in Method S2 in Multimedia Appendix 1, with further details on each assessment tool provided in Method S3 in Multimedia Appendix 1. Diagnoses of MCI and dementia were determined via consensus conference by 3 dementia specialists. Dementia was diagnosed using the *Diagnostic and Statistical Manual of Mental Disorders, Fifth Edition*, criteria [46], which require the presence of cognitive concerns (reported by the individual or a knowledgeable informant), impairment in instrumental activities of daily living (eg, managing money or medications), and objective cognitive deficits. MCI was diagnosed using the modified Petersen criteria [47], which require the presence of cognitive concerns (reported by the individual or a knowledgeable informant), absence of impairment in instrumental activities of daily living, and the presence of objective cognitive deficits. Normal cognition was diagnosed when participants did not meet criteria for dementia or MCI.

### Measures

AD8 [30] is an 8-item, informant-based questionnaire that assesses changes in a participant’s cognition and function over the past few years. For each item, the informant rates whether there has been a change in the participant: 1=yes (a change) and 0=no (no change) or don’t know. Responses to the 8 items are summed to provide a total score, with higher scores reflecting greater cognitive problems. AD8 has been shown to be useful in detecting varying severities of cognitive impairment [37,38]. Informant AD8 has also been previously validated in Singapore [48].

To derive an ultra-brief questionnaire, the study team focused on candidate questions that assess the two key criteria for diagnosing MCI/dementia [46,47]: (1) the presence of cognitive concerns as reported by the individual or a knowledgeable informant; and (2) impairment in instrumental activities of daily living (iADL). The presence of cognitive concerns was evaluated using validated questions related to subjective cognitive decline (SCD), by asking participants or informants: “Do you feel like your (or your family member’s) memory is becoming worse?” and “Are you *worried* that your (or your family member’s) memory is becoming worse?” These 2 sets of questions have been validated in previous studies [49,50] and have been shown to be useful for capturing early symptoms of cognitive decline [51-55].

Impairment in iADL was assessed using the locally validated modified Lawton scale [56], with informants asked about difficulties in various domains (public commuting, grocery shopping, managing money, using the telephone, taking medications, preparing meals, doing housework, and doing laundry). The original responses on the modified Lawton scale included 4 options (ie, unable to do at all, needs some help, needs no help, and never needed to do this), which were collapsed in this study into two options: (1) yes and (2) no/never needed to do this.

In total, 15 candidate questions were considered for the ultra-brief questionnaire: 12 cognition-related items and 3 basic demographic variables (eg, age, sex, and years of education), given their potential correlation with cognition. The exact wording and response options for all 15 items are provided in Table 1.

**Table 1.** A preselected list of 15 question items that are potentially related to cognition.

Question category ^a^	Question item ^a^	Response options
SCD^b^ question—informant	Do you feel like your family member’s memory is becoming worse?	1=yes; 0=no/not sure
Worry about cognition—informant	Are you *worried* that your family member’s memory is becoming worse?	1=yes; 0=no/not sure
SCD question—participant	Do you feel like your memory is becoming worse?	1=yes; 0=no/not sure
Worry about cognition—participant	Are you *worried* that your memory is becoming worse?	1=yes; 0=no/not sure
iADL^c^—commute	Does your family member need help to take public transport or drive a car?	1=yes; 0=no/never needed to do this
iADL—grocery	Does your family member need help to do grocery shopping?	1=yes; 0=no/never needed to do this
iADL—money	Does your family member need help to manage money?	1=yes; 0=no/never needed to do this
iADL—telephone	Does your family member need help to use the telephone?	1=yes; 0=no/never needed to do this
iADL—medications	Does your family member need help to take medications?	1=yes; 0=no/never needed to do this
iADL—meals	Does your family member need help to prepare meals?	1=yes; 0=no/never needed to do this
iADL—housework	Does your family member need help to do housework?	1=yes; 0=no/never needed to do this
iADL—laundry	Does your family member need help to do laundry?	1=yes; 0=no/never needed to do this
Age	What is your family member’s age?	Continuous variable
Sex	What is your family member’s sex?	1=male; 0=female
Years of education	What is your family member’s years of education?	Continuous variable. Count the years of full-time education, starting from elementary/primary school.

a Items in the list were selected to assess the 2 key criteria in the diagnosis of mild cognitive impairment and dementia: (1) the presence of cognitive concerns as reported by the individual or a knowledgeable informant and (2) impairment in iADL [46,47]. The presence of cognitive concerns was evaluated using validated questions related to SCD, given prior literature on the usefulness of SCD to reflect early symptoms of cognitive decline [51-55]. Impairment in iADL was assessed using the locally validated modified Lawton scale [56], with informants asked about difficulties in various domains of iADL. Three basic demographic variables (ie, age, sex, and years of education) were also included, given their potential correlation with cognition.

b SCD: subjective cognitive decline.

c iADL: instrumental activities of daily living.

### Statistical Analyses

The study sample was randomly split into a 70% *training sample* and a 30% *test sample*. The *training sample* was used to develop an ultra-brief questionnaire that best distinguished MCI/dementia from normal cognition, whereas the *test sample* was used to evaluate the actual performance of this questionnaire.

In the *training sample*, a best-subset approach [57] with 5-fold cross-validation was used to select the optimal combination of items from the 15 candidate questions. The best-subset approach is an efficient, computationally intensive method for variable selection [4,20,58,59] in which logistic regression is used to exhaustively evaluate all possible combinations of the candidate questions, identifying models with the lowest prediction errors. Five-fold cross-validation was then used to select the most parsimonious model within 1 SE of the best-performing model. After identifying the optimal model from the best-subset approach, we further refined the model by considering potential inclusions of quadratic terms for continuous variables (eg, age and years of education); quadratic terms with *P*<.05 were incorporated into the final model to improve fit. The final selected model was then applied to the test sample to generate predicted probabilities of cognitive impairment (ie, MCI/dementia), with the model variables constituting the new ultra-brief questionnaire.

Predicted probabilities were computed from logistic regression using the following equation:

Predicted probability=$\frac{e^{Logit}}{1+e^{Logit}}$

where


$$logit=\beta_{0}\;+\;\beta_{1}\cdot \left(Variable\;1\right)\;+\;\beta_{2}\cdot \left(Variable\;2\right)\;+\;\beta_{3}\cdot \left(Variable\;3\right)\;+\;\ldots$$

with each β representing the regression coefficient for its respective variable in the model.

In the *test sample*, the predicted probabilities were used to compute the area under the receiver operating characteristic curve (AUC), thereby assessing the actual performance of the ultra-brief questionnaire in discriminating MCI/dementia from normal cognition. In general, an AUC of 0.7 to 0.8 is considered acceptable discrimination, 0.8 to 0.9 is considered excellent, and more than 0.9 is considered outstanding [60]. Comparisons of AUC were conducted using the nonparametric method proposed by DeLong et al [33,59,61,62], with analyses stratified by education subgroups (ie, ≤10 and >10 y of education based on median split). A 2-cutoff approach [63-67] was adopted for the ultra-brief questionnaire. The first cutoff was chosen to yield high sensitivity and negative predictive value (>80% each) and was used to rule out MCI/dementia (ie, when probability scores fell below the first threshold). The second cutoff was selected for high specificity and positive predictive value (>80% each), identifying those very likely to have MCI/dementia. This 2-cutoff approach is recommended in recent literature [67], as it enhances the performance of cognitive assessment tools [63-66], reduces the effects of prevalence on tool performance [64], and allows prioritization of scarce health care resources for individuals who truly require further cognitive assessments [63].

As a secondary analysis, the performance of the ultra-brief questionnaire was also evaluated for distinguishing dementia from nondementia. Additionally, 2 sensitivity analyses were conducted in the test sample to evaluate the robustness of results when the prevalence of MCI/dementia was readjusted to reflect the average prevalence in most communities:

- Prevalence of MCI/dementia was artificially readjusted to 20% based on prior meta-analytic findings that community prevalence is ~15% for MCI [68-70] and ~5% for dementia [71-73]. Readjustment of prevalence was done by randomly selecting a subset of participants with MCI and normal cognition—for each participant with dementia, 3 participants with MCI and 16 participants with normal cognition were randomly selected (ie, so that the final dataset corresponded to 5% prevalence for dementia and 15% prevalence for MCI).
- Prevalence of MCI/dementia was artificially readjusted to 35% based on prior meta-analytic findings that community prevalence could be as high as ~25% for MCI [69,70] and ~10% for dementia [72,73]. Readjustment of prevalence was done by randomly selecting only a subset of participants with MCI and normal cognition—for each participant with dementia, 2.5 participants with MCI and 6.5 participants with normal cognition were randomly selected (ie, so that the final dataset corresponded to 10% prevalence for dementia and 25% prevalence for MCI).

The best-subset approach was performed with the “bestglm” [57] package in R (version 4.4.0; R Foundation for Statistical Computing). All remaining analyses were conducted in Stata (version 18; StataCorp LLC). No a priori sample size calculation was performed; the final sample size was determined pragmatically based on participants recruited between March 2022 and September 2024. Post hoc power analyses confirmed that the test sample provided robust power (90%) to distinguish MCI/dementia from normal cognition (α=.05, two-sided test). Power was also reasonably sufficient for participants with ≤10 years of education (72%) but was limited in those with >10 years of education (19%) due to fewer positive cases. The power calculations were conducted using PASS software (version 15.0.5; NCSS, LLC) and the Hanley and McNeil formula [74], with further details provided in MethodS4 in Multimedia Appendix 1.

### Ethical Considerations

The study received ethical approval from the SingHealth Centralized IRB of Singapore (reference number: 2021/2590). Informed consent was obtained from all participants. Before obtaining informed consent, the mental capacity of participants was briefly assessed in accordance with the Mental Capacity Act of Singapore [75]. In the event of concerns about mental capacity, informed consent by proxy was then obtained from the legally authorized next-of-kin. Participants who completed the research assessments received Singapore Dollar $80 as compensation for their time, inconvenience and transportation costs.

## Results

### Overview

A total of 1856 participants were included, of whom 255 (13.7%) had MCI/dementia. Participant characteristics are presented in Table 2, with a median age of 72 years and a median education of 10 years (range 0-23 y). Corresponding informant characteristics are provided in Table S1 in Multimedia Appendix 1, with informants primarily comprising spouses (897/1856, 48.3%) and children (506/1856, 27.3%). The sample was randomly split into a *training sample* (1299/1856, 70%) and a *test sample* (557/1856, 30%). Table S2 in Multimedia Appendix 1 shows that the training and test samples had comparable demographic characteristics (*P*>.05 across covariates).

**Table 2.** Characteristics of the study participants.

Variable	Overall sample (n=1856)	Normal cognition (n=1601)	MCI^a^ (n=207)	Dementia (n=48)	*P* value ^b^
Age, median (IQR) [range]	72 (68 to 76) [65 to 101]	71 (68 to 75) [65 to 93]	74 (70 to 79) [65 to 91]	80 (76 to 82) [66 to 101]	<.001
Years of education, median (IQR) [range]	10 (9 to 13) [0 to 23]	10 (10 to 13) [0 to 23]	10 (6 to 12) [0 to 21.5]	10 (2 to 10) [0 to 17]	<.001
Sex (male), n (%)	688 (37.1)	572 (35.7)	101 (48.8)	15 (31.2)	<.001
Ethnicity, n (%)					.39
Chinese	1735 (93.5)	1504 (93.9)	188 (90.8)	43 (89.6)	
Malay/Indian	96 (5.2)	77 (4.8)	15 (7.2)	4 (8.3)	
Eurasian/others	25 (1.3)	20 (1.2)	4 (1.9)	1 (2.1)	
MoCA^c^ total score, median (IQR)	26 (24 to 28)	27 (25 to 28)	21 (17 to 24)	14 (9 to 19)	<.001
NTB^d^ Global Z-scores, median (IQR)	−0.2 (−0.6 to 0.1)	−0.1 (−0.4 to 0.2)	−1.0 (−1.3 to 0.7)	−1.6 (−2.1 to 1.2)	<.001
Global CDR^e^, n (%)					<.001
0	1570 (84.6)	1557 (97.3)	13 (6.3)	0 (0.0)	
0.5	255 (13.7)	44 (2.7)	194 (93.7)	17 (35.4)	
1	22 (1.2)	0 (0.0)	0 (0.0)	22 (45.8)	
2	8 (0.4)	0 (0.0)	0 (0.0)	8 (16.7)	
3	1 (0.1)	0 (0.0)	0 (0.0)	1 (2.1)	

a MCI: mild cognitive impairment.

b Test of difference across diagnoses: chi-square test for categorical variables and Kruskal-Wallis test for continuous variables.

c MoCA: Montreal Cognitive Assessment.

d NTB: neuropsychological battery.

e CDR: clinical dementia rating.

### Development of the Ultra-Brief Questionnaire

**Figure 1.**
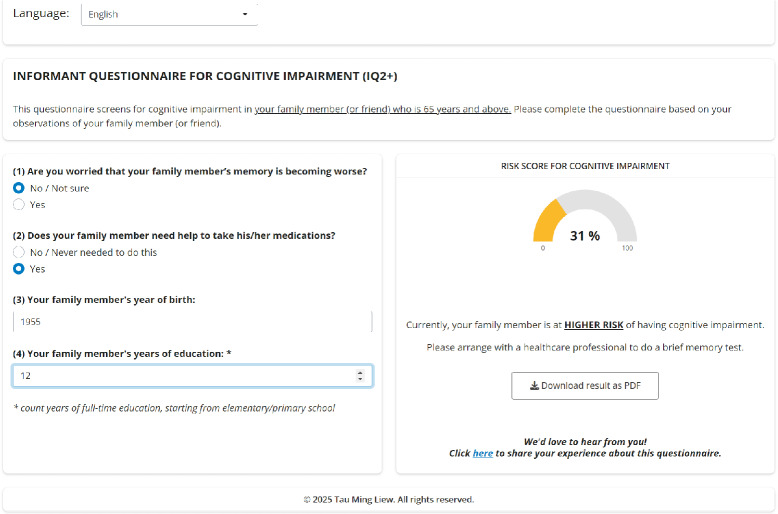
A sample screenshot of the interactive web-based calculator. The web-based calculator can be accessed at [76].

Table 3 presents the top models identified through the exhaustive search method in the *training sample* (n=1299). *iADL–medications* and *worry about cognition–informant* were among the most useful items in detecting MCI/dementia, whereas *iADL–meals* and *iADL–grocery* were among the least useful. Following 5-fold cross-validation, the model with 4 items was identified as the most parsimonious among the top models (Figure S1 in Multimedia Appendix 1). This model included 2 informant questions (*iADL–medications* and *worry about cognition–informant*), plus 2 demographic variables (*age* and *years of education*). These items were then selected to constitute the new ultra-brief questionnaire, henceforth denoted as IQ2+ (Informant Questionnaire for cognitive impairment–2 items plus demographics). Further model refinement considered the inclusion of quadratic terms for *age* and *years of education*, resulting in the addition of a quadratic term for years of education (*P*=.004) in the final model. Thus, the final IQ2+ model comprised five predictors: iADL–medications, worry about cognition–informant, age, years of education, and the quadratic term for years of education. Responses from the IQ2+ questionnaire can be converted to predicted probabilities using an interactive web-based calculator we have created [76]. A screenshot of the calculator is shown in Figure 1.

**Table 3.** The top models that best discriminate mild cognitive impairment and dementia from normal cognition (as identified by the best-subset approach) in the training sample (n=1299).

Question items, rearranged by their usefulness in detecting MCI^a^/dementia^b^	Number of items in the top models														
	1	2	3	4^c^	5	6	7	8	9	10	11	12	13	14	15
iADL^d^—medications	●	●	●	●	●	●	●	●	●	●	●	●	●	●	●
Worry about cognition—informant	N/A^e^	●	●	●	●	●	●	●	●	●	●	●	●	●	●
Age	N/A	N/A	●	●	●	●	●	●	●	●	●	●	●	●	●
Years of education	N/A	N/A	N/A	●	●	●	●	●	●	●	●	●	●	●	●
Sex	N/A	N/A	N/A	N/A	●	●	●	●	●	●	●	●	●	●	●
iADL—money	N/A	N/A	N/A	N/A	N/A	●	●	●	●	●	●	●	●	●	●
SCD^f^ question—informant	N/A	N/A	N/A	N/A	N/A	N/A	●	●	●	●	●	●	●	●	●
iADL—commute	N/A	N/A	N/A	N/A	N/A	N/A	N/A	●	●	●	●	●	●	●	●
iADL—telephone	N/A	N/A	N/A	N/A	N/A	N/A	N/A	N/A	●	●	●	●	●	●	●
Worry about cognition—participant	N/A	N/A	N/A	N/A	N/A	N/A	N/A	N/A	N/A	●	●	●	●	●	●
iADL—laundry	N/A	N/A	N/A	N/A	N/A	N/A	N/A	N/A	N/A	N/A	●	●	●	●	●
SCD question—participant	N/A	N/A	N/A	N/A	N/A	N/A	N/A	N/A	N/A	N/A	N/A	●	●	●	●
iADL—housework	N/A	N/A	N/A	N/A	N/A	N/A	N/A	N/A	N/A	N/A	N/A	N/A	●	●	●
iADL—grocery	N/A	N/A	N/A	N/A	N/A	N/A	N/A	N/A	N/A	N/A	N/A	N/A	N/A	●	●
iADL—meals	N/A	N/A	N/A	N/A	N/A	N/A	N/A	N/A	N/A	N/A	N/A	N/A	N/A	N/A	●

a MCI/dementia: mild cognitive impairment or dementia.

b A full description of each item is available in Table 1. Briefly, items in the list were selected to assess the 2 key criteria in the diagnosis of mild cognitive impairment and dementia: (1) the presence of cognitive concerns as reported by the individual or a knowledgeable informant and (2) impairment in iADL [46,47]. The presence of cognitive concerns was evaluated using validated questions related to SCD, given prior literature on the usefulness of SCD to reflect early symptoms of cognitive decline [51-55]. Impairment in iADL was assessed using the locally validated modified Lawton scale [56], with informants asked about difficulties in various domains of iADL. Three basic demographic variables (ie, age, sex, and years of education) were also included, given their potential correlation with cognition.

c The 4-item model was identified as the most parsimonious among the top models. Further details on model selection are available in Figure S1 in Multimedia Appendix 1.

d iADL: instrumental activities of daily living.

e N/A: not applicable.

f SCD: subjective cognitive decline

### Performance of the Ultra-brief Questionnaire

Table 4 presents the AUC results for IQ2+ and AD8 in the *test sample* (n=557). Predicted probabilities from the new IQ2+ demonstrated excellent performance in distinguishing MCI/dementia from normal cognition (AUC 85.3%, 95% CI 80.4%‐90.2%), which was significantly better (*P*=.003) than a nested baseline model (comprising age, years of education, and the quadratic term of years of education; AUC 78.0%, 95% CI 72.6%‐83.4%). In contrast, AD8 had an AUC of 76.1% (95% CI 69.6%‐82.6%), which was not significantly different (*P>*.99) from that of the baseline model. For the detection of dementia, both IQ2+ and AD8 had AUCs >90%, which were significantly higher (*P*<.05) than the baseline model (83.2%). AUC results remained largely similar across education subgroups and in the 2 sensitivity analyses where the prevalence of MCI/dementia was increased to reflect average prevalence in most communities (ie, 20% [68-73] and 35% [69,70,72,73], respectively).

**Table 4.** Performance of IQ2+ for detecting cognitive impairment in the test sample (n=557) and a comparison with the performance of AD8.

Assessment tool	All education subgroups		≤10 y of education^a^		>10 y of education^a^	
	AUC^b^, % (95% CI)	*P* value^c^	AUC, % (95% CI)	*P* value^c^	AUC, % (95% CI)	*P* value^c^
Detection of MCI^d^/dementia						
Baseline model (age and education)^e^	78.0 (72.6‐83.4)	Ref^f^	76.7 (69.9‐83.5)	Ref	74.8 (65.2‐84.5)	Ref
IQ2+^g^	85.3 (80.4‐90.2)	.003	84.5 (78.6‐90.4)	.015	83.3 (72.5‐94.0)	.095
AD8^h^	76.1 (69.6‐82.6)	1.000	75.5 (68.0‐83.0)	1.000	76.8 (62.7‐91.0)	1.000
Detection of dementia						
Baseline model (age and education)^e^	83.2 (72.8‐93.6)	Ref	77.8 (64.5‐91.2)	Ref	94.1 (90.2‐97.9)	Ref
IQ2+	96.7 (92.8‐100)	.035	95.1 (89.6‐100)	.034	99.8 (99.2‐100)	.003
AD8	99.4 (98.8‐99.9)	.005	99.6 (99.1‐100)	.003	99.1 (97.8‐100)	.059
Sensitivity analysis 1 (prevalence of MCI/dementia=20%)^i^						
Detection of MCI/dementia						
Baseline model (age and education)^e^	74.8 (68.3‐81.3)	Ref	73.8 (65.7‐81.9)	Ref	70.1 (58.4‐81.7)	Ref
IQ2+	85.2 (79.6‐90.8)	<.001	84.3 (77.4‐91.2)	.005	83.6 (72.1‐95.0)	.023
AD8	77.1 (70.1‐84.2)	1.000	75.9 (67.6‐84.2)	1.000	79.1 (64.7‐93.5)	.525
Detection of dementia						
Baseline model (age and education)^e^	81.5 (70.7‐92.3)	Ref	76.4 (62.9‐90.0)	Ref	92.5 (87.7‐97.4)	Ref
IQ2+	96.5 (92.1‐100)	.023	94.8 (88.5‐100)	.026	100 (100‐100)	.005
AD8	99.1 (98.3‐100)	.003	99.5 (98.9‐100)	.002	98.6 (96.5‐100)	.069
Sensitivity analysis 2 (prevalence of MCI/dementia=35%) ^j^						
Detection of MCI/dementia						
Baseline model (age and education)^e^	77.0 (69.3‐84.8)	Ref	75.6 (65.8‐85.5)	Ref	73.2 (58.0‐88.3)	Ref
IQ2+	85.1 (78.5‐91.6)	.031	83.8 (75.7‐91.9)	.135	82.4 (67.6‐97.1)	.280
AD8	73.4 (65.0‐81.9)	1.000	73.5 (63.5‐83.4)	1.000	73.6 (56.3‐91.0)	1.000
Detection of dementia						
Baseline model (age and education)^e^	80.1 (68.5‐91.7)	Ref	73.6 (58.7‐88.5)	Ref	95.8 (90.6‐100)	Ref
IQ2+	94.4 (89.3‐99.5)	.044	91.8 (84.4‐99.2)	.046	99.2 (96.8‐100)	.307
AD8	98.8 (97.5‐100)	.004	99.2 (98.1‐100)	.002	98.3 (95.2‐100)	.893

a Education subgroups were stratified based on median split. This subgroup analysis has reduced statistical power (see Method S4 in Multimedia Appendix 1 for details) and is exploratory in nature.

b AUC: area under the receiver operating characteristic curve.

c *P* values were based on comparisons of AUC using the nonparametric method proposed by DeLong et al [61]. *P*<.05 indicates significant difference in AUC between the baseline model and the respective assessment tools. *P* values were Bonferroni-adjusted to minimize the risk of type 1 error in the context of multiple testing.

d MCI/dementia: mild cognitive impairment or dementia.

e This baseline model was provided mainly for comparison purposes, by omitting IQ2+’s 2 core questions (ie, *assistance with medications* and *worry about cognition*) to examine the incremental utility of the 2 core questions beyond those provided by the demographic information of age and education. The baseline model was generated in the training sample using a logistic model with the dependent variable of MCI/dementia and with the independent variables of age, years of education, and the quadratic term of years of education. This baseline model was then applied to the test sample to generate predicted probabilities of MCI/dementia, with the predicted probabilities used for AUC comparisons.

f Ref: reference.

g IQ2+: the Informant Questionnaire for cognitive impairment–2 items (plus demographics).

h AD8: the 8-item Informant Interview to Differentiate Aging and Dementia.

i Prevalence of MCI/dementia was readjusted to 20% in the test sample based on prior meta-analytic findings that community prevalence was ~15% for MCI and ~5% for dementia. In the test sample, a subset of participants with MCI and dementia was randomly selected to readjust the prevalence in the dataset (see Methods section for further details). The resulting dataset comprised 256 participants with normal cognition (80%), 48 participants with MCI (15%), and 16 participants with dementia (5%).

j Prevalence of MCI/dementia was readjusted to 35% in the test sample based on prior meta-analytic findings that community prevalence could be as high as ~25% for MCI and ~10% for dementia. In the test sample, a subset of participants with MCI and dementia was randomly selected to readjust the prevalence in the dataset (see Methods section for further details). The resulting dataset comprised 104 participants with normal cognition (65%), 40 participants with MCI (25%), and 16 participants with dementia (10%).

### Optimal Cutoffs of the Ultra-Brief Questionnaire

**Figure 2.**
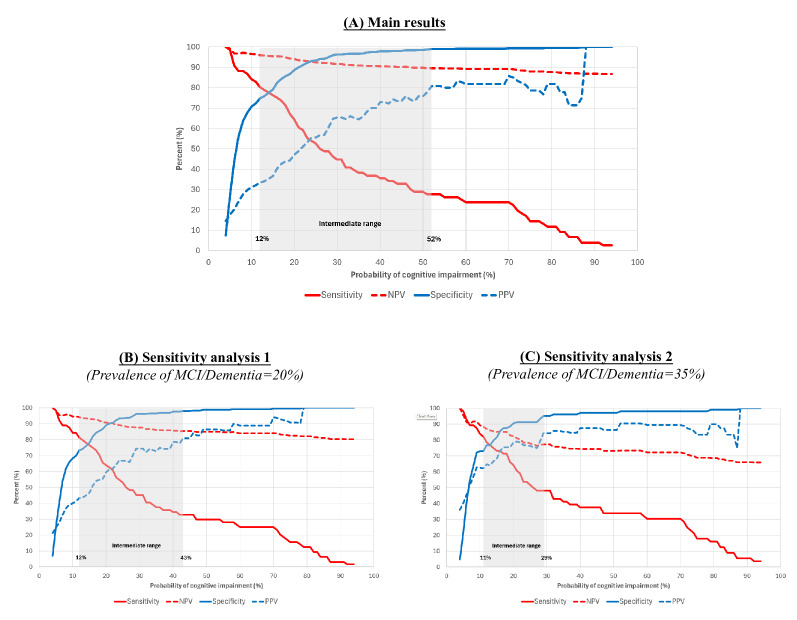
Plot of sensitivity, specificity, NPV, and PPV based on probabilities of cognitive impairment generated from IQ2+ in the test sample (n=557). This plot is intended to demonstrate the 2-cutoff approach. The lower cutoff identifies sensitivity and NPV (red lines), which are >80% each and are used to rule out MCI/dementia (when probability scores fall below this threshold). The upper cutoff identifies specificity and PPV (blue lines), which are >80% each, and are used to rule in MCI/dementia (when probability scores exceed this threshold). The grayed area (demarcated by the lower and upper cutoffs) represents the intermediate range, identifying those who may warrant further assessment. Plot (A) was based on the main results from all the test samples (n=577). Plot (B) was based on results from sensitivity analysis 1, whereby the prevalence of MCI/dementia was readjusted to 20% in the test sample based on prior meta-analytic findings that community prevalence was ~15% for MCI and ~5% for dementia (in the test sample, a subset of participants with MCI and dementia were randomly selected in the test sample to readjust the prevalence in the dataset; see Methods section for further details). Plot (C) was based on results from sensitivity analysis 2, whereby the prevalence of MCI/dementia was readjusted to 35% in the test sample based on prior meta-analytic findings that community prevalence could be as high as ~25% for MCI and ~10% for dementia (in the test sample, a subset of participants with MCI and dementia were randomly selected in the test sample to readjust the prevalence in the dataset; see Methods section for further details). IQ2+: the Informant Questionnaire for Cognitive Impairment–2 Items (plus demographics); MCI/dementia: mild cognitive impairment or dementia; NPV: negative predictive value; PPV: positive predictive value.

Test statistics for IQ2+ are plotted in Figure 2A. Adopting a 2-cutoff approach, the lower cutoff (probability ≥12%) had 80.3% sensitivity and 96.0% negative predictive value and was used to rule out MCI/dementia (for individuals with probability scores below the cutoff), whereas the upper cutoff (probability ≥52%) had 99.0% specificity and 80.8% positive predictive value and identified those likely to have MCI/dementia (ie, to rule in MCI/dementia). These 2 cutoffs provide an intermediate range between them (grayed area in Figure 2A), identifying those who warrant further assessment. The optimal cutoffs varied slightly with changing prevalence of MCI/dementia, as shown in Figure 2B and 2C. Detailed results on test statistics for IQ2+ are also available in Tables S3–S5 in Multimedia Appendix 1.

### Result Interpretation for the Ultra-Brief Questionnaire

**Figure 3.**
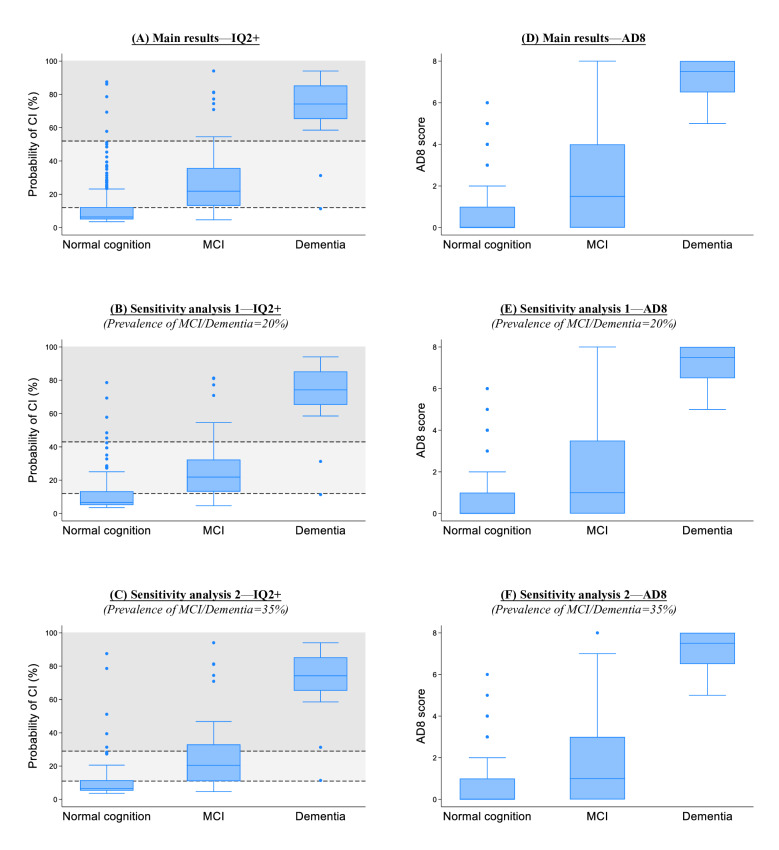
Box plots showing the distribution of IQ2+ and AD8 in the test sample (n=577). Plots (A) and (B) were based on the main results from all the test samples (n=577). Plots (C) and (D) were based on results from sensitivity analysis 1, whereby the prevalence of MCI/dementia was readjusted to 20% in the test sample based on prior meta-analytic findings that community prevalence was ~15% for MCI and ~5% for dementia (in the test sample, a subset of participants with MCI and dementia were randomly selected in the test sample to readjust the prevalence in the dataset; see Methods section for further details). Plots (E) and (F) were based on results from sensitivity analysis 2, whereby the prevalence of MCI/dementia was readjusted to 35% in the test sample based on prior meta-analytic findings that community prevalence could be as high as ~25% for MCI and ~10% for dementia (in the test sample, a subset of participants with MCI and dementia were randomly selected in the test sample to readjust the prevalence in the dataset; see Methods section for further details). In plots (B), (D), and (F), the 2 horizontal dashed lines represent the 2 optimal cutoffs for IQ2+. The lower cutoff has high sensitivity and negative predictive value (>80% each) and is used to rule out MCI/dementia when probability scores fall below this threshold (as shown by the white region). The upper cutoff has high specificity and positive predictive value (>80% each) and identifies individuals likely to have MCI/dementia (when probability scores exceed this threshold, as shown by the dark gray region). The light gray region (demarcated by the lower and upper cutoffs) represents the intermediate range, identifying those who may warrant further assessment. AD8: the 8-item Informant Interview to Differentiate Aging and Dementia; IQ2+: the Informant Questionnaire for Cognitive Impairment–2 Items (plus demographics); CI: cognitive impairment; MCI/dementia: mild cognitive impairment or dementia.

The 2 cutoffs of IQ2+ effectively identified three risk categories for cognitive impairment: (1) less likely to have cognitive impairment, (2) higher risk of cognitive impairment, and (3) likely to have cognitive impairment. These 3 categories, along with their cross-tabulation with the final diagnoses, are presented in Table 5. In the first category (ie, less likely to have cognitive impairment), 88% to 96% of individuals had normal cognition. In the second category (ie, higher risk of cognitive impairment), 25% to 45% of individuals were diagnosed with MCI. In the third category (ie, likely to have cognitive impairment), 81% to 84% of the individuals had MCI/dementia, with a large proportion having dementia (47%-54%). Distinctions between these 3 risk categories of IQ2+ are also visible in the box plots in Figure 3A–3C. The first category (white region with probability scores below the lower cutoff) identified those with normal cognition, the third category (dark gray region with probability scores above the upper cutoff) identified almost all individuals with dementia, and the second category (light gray region between the lower and upper cutoffs) mostly captured those with MCI. In contrast, AD8 showed poorer discrimination between normal cognition and MCI, with discernible floor effects in the normal cognition and MCI groups as seen in the box plots in Figure 3D–3F. This is also reflected in the test statistics of AD8 (as presented in Tables S6–S8 in Multimedia Appendix 1), which demonstrated low sensitivity across its cutoff scores (with maximum sensitivity of 69.6%‐73.4%).

**Table 5.** Cross-tabulation between the output from IQ2+ and the final diagnosis in the test sample (n=557).

Result from IQ2+^a^^b^	Final diagnosis		
	Normal cognition	MCI^c^	Dementia
Less likely to have CI, n (%)^d^	360 (96.0)	14 (3.7)	1 (0.3)
Higher risk of CI, n (%)^d^	116 (74.4)	39 (25.0)	1 (0.6)
Likely to have CI, n (%) ^d^	5 (19.2)	7 (26.9)	14 (53.8)
Sensitivity analysis 1 (prevalence of MCI/dementia=20%)^e^			
Less likely to have CI, n (%)^f^	188 (94.0)	11 (5.5)	1 (0.5)
Higher risk of CI, n (%)^f^	63 (67.0)	30 (31.9)	1 (1.1)
Likely to have CI, n (%)^f^	5 (19.2)	7 (26.9)	14 (53.8)
Sensitivity analysis 2 (Prevalence of MCI/dementia=35%)^g^			
Less likely to have CI, n (%)^h^	76 (88.0)	10 (12.0)	0 (0.0)
Higher risk of CI, n (%)^h^	23 (55.0)	18 (43.0)	1 (2.0)
Likely to have CI, n (%)^h^	5 (16.0)	12 (38.0)	15 (47.0)

a IQ2+, the Informant Questionnaire for cognitive impairment–2 items (plus demographics).

b Two-cutoff approach was adopted for IQ2+. The lower cutoff has high sensitivity and negative predictive value (>85% respectively) and is used to rule out MCI/dementia (for individuals with probability scores below the cutoff). The upper cutoff has high specificity and positive predictive value (>85% respectively) and identifies those who are likely to have MCI/dementia. These 2 cutoffs provide an intermediate range between them, identifying those who may be at higher risk and require further monitoring or assessment.

c MCI: mild cognitive impairment.

d Probability cutoff for the main results (ie, prevalence of MCI/dementia=14%):<12% (less likely to have CI), 12% to 51% (higher risk of CI), and ≥52% (likely to have CI).

e Prevalence of MCI/dementia was readjusted to 20% in the test sample based on prior meta-analytic findings that community prevalence was ~15% for MCI and ~5 % for dementia. In the test sample, a subset of participants with MCI and dementia was randomly selected to readjust the prevalence in the dataset (see Methods section for further details).

f Probability cutoff for the first sensitivity analysis (ie, prevalence of MCI/dementia=20%):<12% (less likely to have CI), 12% to 42% (higher risk of CI), and ≥43% (likely to have CI).

g Prevalence of MCI/dementia was readjusted to 35% in the test sample based on prior meta-analytic findings that community prevalence could be as high as ~25% for MCI and ~10% for dementia. In the test sample, a subset of participants with MCI and dementia was randomly selected to readjust the prevalence in the dataset (see Methods section for further details).

h Probability cutoff for the second sensitivity analysis (ie, prevalence of MCI/dementia=35%):<11% (less likely to have CI), 11% to 28% (higher risk of CI), and ≥29% (likely to have CI).

## Discussion

### Principal Findings

In this study, we developed and validated an ultra-brief informant questionnaire (IQ2+) for the detection of cognitive impairment in literacy-diverse communities. Using a rigorous best-subset approach with cross-validation, we identified a parsimonious 4-item model that was most useful in detecting MCI/dementia, comprising 2 informant-based questions (*assistance with medications* and *worry about cognition*), along with *age* and *years of education*. In the independent test sample, IQ2+ achieved an AUC of 85.3%, outperforming the widely used AD8 (AUC=76.1%) and a baseline demographic model (AUC=78.0%). In contrast, AD8 was not significantly better than the baseline demographic model. The robust performance of IQ2+ was consistent across education subgroups and under varying prevalence scenarios, highlighting its potential utility for case finding in diverse community settings. Adopting 2 optimal cutoffs [63-67], IQ2+ demonstrated high sensitivity, specificity, negative predictive value, and positive predictive value.

### Interpretation of Findings

Despite being widely recommended [26,39-42], the AD8 performed poorly in detecting MCI/dementia and was not substantially better than a basic demographic model comprising age and education. This finding aligns with recent literature, which demonstrates AD8’s lower performance for detecting early cognitive impairment (AUC 61%‐69%) compared to its performance in clear-cut dementia cases (AUC 89%‐93%) [48,77]. AD8 was originally developed for the purpose of detecting dementia [30], and its items tend to focus on identifying conspicuous changes in iADL (ie, hallmarks of the onset of dementia) [46]. As a result, AD8 may possibly not be as sensitive or consistent in detecting subtle changes associated with early cognitive impairment [30,78]. In contrast, the 2 informant questions in IQ2+ may plausibly be more sensitive to early cognitive changes. Worry about cognition—especially when reported by a knowledgeable informant—has been shown to be an early symptom of cognitive decline [51]. This item requires informants to compare current abilities with previous premorbid abilities, making it more likely to detect subtle cognitive decline over time. It also prompts informants to apply a “threshold” to decide whether they are worried about the cognitive decline, which serves as a filter to identify individuals with meaningful changes. Similarly, the ability to manage one’s medications is a complex task that possibly involves multiple cognitive domains [16], such as language (for understanding medication instructions), executive function (for scheduling medication intake and problem-solving when medications are not taken on schedule), and memory (for tracking medication intake). A subtle decline in any of these domains may potentially manifest as increasing difficulty in managing medications independently, serving as an early symptom of cognitive decline [79].

### Implications

At the population level, IQ2+ can serve as a quick and efficient risk stratification tool, facilitating appropriate triage for further assessment. As summarized in Table 6, low-risk individuals (<12% probability of MCI/dementia) may be reassured and advised to repeat the test in 3 to 5 years or if circumstances change (eg, appearance of new symptoms). Intermediate-risk individuals (~25%‐45% probability of MCI/dementia) can be directed to further assessments using brief cognitive tests [4,32-34] to provide more conclusive evidence of cognitive impairment [27]. High-risk individuals (>80% probability of MCI/dementia) may benefit from direct referral to memory clinics for further clinical management. This approach, as summarized in Table 6, aligns with the 2-stage strategy for active case finding of cognitive impairment (ie, subjective reports, followed by brief cognitive tests) [27,28]. It offers a scalable approach to case finding in large populations by conserving resources for cognitive testing and reserving them for individuals who truly need further verification [27]. To ensure that IQ2+ remains useful in diverse populations, its optimal cutoffs can also be adjusted depending on the prevalence of MCI/dementia in different populations (as shown in Figure 1), thus providing a more tailored solution for identifying individuals at varying risk levels.

**Table 6.** Potential clinical implications based on the result from IQ2+.

Result from IQ2+^a^	Risk communication	Potential implications
Less likely to have CI^b^	<12% chance to have CI	Repeat IQ2+ in 3 to 5 years or when circumstances change (eg, appearance of new symptoms)
Higher risk of CI	~25% to 45% chance to have CI	Arrange with health care professionals to do brief cognitive tests
Likely to have CI	>80% chance to have CI	Referral to memory clinic for further assessment and management

a IQ2+: the Informant Questionnaire for cognitive impairment–2 items (plus demographics).

b CI: cognitive impairment.

Essentially, IQ2+ may have 2 plausible use cases in the community. First, it allows family members who have concerns about a loved one’s cognition to complete the questionnaire online (accessible at [76]), providing an immediate result with risk score and a brief interpretation of its implications. The results can also be downloaded as a PDF file for further discussion with health care providers. This approach mirrors a well-established practice in the field of diabetes mellitus, where the public is encouraged to complete an online risk test [80] as an initial screening to determine the need for further diagnostic tests [81]. By enabling self-appraisal, this use case of IQ2+ leverages family members for efficient case finding and empowers them to proactively support the brain health of older persons.

Second, IQ2+ can also be used by health care workers who routinely conduct comprehensive geriatric assessments in the community. Since 2019, the WHO has advocated for routine assessment of intrinsic capacity among community-dwelling older persons, which requires comprehensive evaluations of cognition, mobility, nutrition, hearing, vision, and mood [29]. Given the extensive range of components to cover, it can be challenging to include routine cognitive testing in all geriatric assessments. IQ2+, which can be completed in <1 minute, is well-suited to be embedded within initial geriatric assessments to prioritize individuals who require further cognitive testing. Given its excellent performance across education subgroups (Table 4), IQ2+ offers a practical tool that may potentially be broadly implemented in diverse populations, including in underserved populations in LMICs, which currently have the largest number of undiagnosed cognitive impairment [82]. This is also in line with the 2024 Lancet Commission’s call to address the unmet need for cognitive screening tools that are also suited for individuals with lower literacy in LMICs [35].

### Limitations

Several limitations are notable. First, IQ2+ was developed for older individuals aged ≥65 years. It is unclear whether IQ2+ would be useful for individuals aged <65 years. Second, IQ2+ would benefit from further validations in other cultures and languages, as cultural differences may affect how informants respond to its items and could potentially modify its performance in different settings. Third, IQ2+ requires the use of a web-based calculator to determine the probability of cognitive impairment. This approach leverages technology to automate test scoring, result visualization, and interpretation. However, it may limit accessibility compared to pen-and-paper tests that use raw scores as a cutoff (eg, AD8), particularly in settings with limited access to technology. Fourth, one of the core questions of IQ2+ involves difficulty managing medications. This item was identified as the most useful item in our exhaustive search (Table 3) and has some face validity (as it detects subtle changes in a complex task of managing medications [16,79], and the need to take regular medications may also be a proxy for higher risk due to the presence of chronic diseases). However, this question may be less useful among healthier older individuals who do not need to take medications regularly. Fifth, although IQ2+ can be a useful case finding tool, it is not intended to replace comprehensive clinical and neuropsychological assessments, which provide more definitive diagnoses as well as granular information on specific cognitive deficits [20,83,84].

### Conclusions

Using rigorous methodology, this study developed an ultra-brief questionnaire that enables untrained laypersons to screen for cognitive impairment in <1 minute. Despite its brevity, the questionnaire demonstrated excellent performance in detecting MCI/dementia, outperforming the well-established AD8. The questionnaire can be completed by members of the public who have concerns about a family member’s cognition (accessible at [76]) or embedded within community geriatric assessments to prioritize cognitive testing. Its excellent performance was consistent across education subgroups and varying prevalence scenarios, supporting its utility for case finding in diverse populations, including underserved communities and LMICs.

## Supplementary material

10.2196/72963 Multimedia Appendix 1 Additional information on methodology and results.

## Abbreviations

AD8: 8-item Informant Interview to Differentiate Aging and Dementia
AUC: area under the receiver operating characteristic curve
iADL: instrumental activities of daily living
IQ2+: Informant Questionnaire for cognitive impairment–2 items plus demographics
LMIC: lower- and middle-income country
MCI/dementia: mild cognitive impairment or dementia
SCD: subjective cognitive decline
WHO: World Health Organization

## References

[1] (2012). Dementia: A Public Health Priority.

[2] Lang L, Clifford A, Wei L (2017). Prevalence and determinants of undetected dementia in the community: a systematic literature review and a meta-analysis. BMJ Open.

[3] Liu Y, Jun H, Becker A, Wallick C, Mattke S (2024). Detection rates of mild cognitive impairment in primary care for the United States Medicare population. J Prev Alzheimers Dis.

[4] Liew TM (2019). A 4-item case-finding tool to detect dementia in older persons. J Am Med Dir Assoc.

[5] Burns A, Iliffe S (2009). Dementia. BMJ.

[6] Ying J, Yap P, Gandhi M, Liew TM (2018). Iterating a framework for the prevention of caregiver depression in dementia: a multi-method approach. Int Psychogeriatr.

[7] Liew TM (2021). Neuropsychiatric symptoms in early stage of Alzheimer’s and non-Alzheimer’s dementia, and the risk of progression to severe dementia. Age Ageing.

[8] Liew TM, Lee CS (2019). Reappraising the efficacy and acceptability of multicomponent interventions for caregiver depression in dementia: the utility of network meta-analysis. Gerontologist.

[9] Ang LC, Malhotra R, Roy Chowdhury A, Liew TM (2025). Pre-and post-COVID-19 trends related to dementia caregiving on Twitter. Sci Rep.

[10] Thyrian JR, Hertel J, Wucherer D (2017). Effectiveness and safety of dementia care management in primary care: a randomized clinical trial. JAMA Psychiatry.

[11] Vickrey BG, Mittman BS, Connor KI (2006). The effect of a disease management intervention on quality and outcomes of dementia care: a randomized, controlled trial. Ann Intern Med.

[12] Cepoiu-Martin M, Tam-Tham H, Patten S, Maxwell CJ, Hogan DB (2016). Predictors of long-term care placement in persons with dementia: a systematic review and meta-analysis. Int J Geriatr Psychiatry.

[13] Jennings LA, Laffan AM, Schlissel AC (2019). Health care utilization and cost outcomes of a comprehensive dementia care program for Medicare beneficiaries. JAMA Intern Med.

[14] Spijker A, Vernooij-Dassen M, Vasse E (2008). Effectiveness of nonpharmacological interventions in delaying the institutionalization of patients with dementia: a meta-analysis. J Am Geriatr Soc.

[15] Bott NT, Sheckter CC, Yang D (2019). Systems delivery innovation for Alzheimer disease. Am J Geriatr Psychiatry.

[16] Elliott RA, Goeman D, Beanland C, Koch S (2015). Ability of older people with dementia or cognitive impairment to manage medicine regimens: a narrative review. Curr Clin Pharmacol.

[17] Persson S, Saha S, Gerdtham UG, Toresson H, Trépel D, Jarl J (2022). Healthcare costs of dementia diseases before, during and after diagnosis: longitudinal analysis of 17 years of Swedish register data. Alzheimers Dement.

[18] Chay J, Koh WP, Tan KB, Finkelstein EA (2024). Healthcare burden of cognitive impairment: evidence from a Singapore Chinese health study. Ann Acad Med Singap.

[19] Liew TM (2025). Distinct trajectories of subjective cognitive decline before diagnosis of neurocognitive disorders: longitudinal modelling over 18 years. J Prev Alzheimers Dis.

[20] Liew TM (2019). Developing a brief neuropsychological battery for early diagnosis of cognitive impairment. J Am Med Dir Assoc.

[21] Ngandu T, Lehtisalo J, Solomon A (2015). A 2 year multidomain intervention of diet, exercise, cognitive training, and vascular risk monitoring versus control to prevent cognitive decline in at-risk elderly people (FINGER): a randomised controlled trial. Lancet.

[22] Baker LD, Espeland MA, Whitmer RA (2025). Structured vs self-guided multidomain lifestyle interventions for global cognitive function: the US POINTER randomized clinical trial. JAMA.

[23] van Dyck CH, Swanson CJ, Aisen P (2023). Lecanemab in early Alzheimer's disease. N Engl J Med.

[24] Sims JR, Zimmer JA, Evans CD (2023). Donanemab in early symptomatic Alzheimer disease: the TRAILBLAZER-ALZ 2 randomized clinical trial. JAMA.

[25] Prince M, Bryce R, Ferri C (2011). World alzheimer report 2011: the benefits of early diagnosis and intervention. https://www.alzint.org/u/WorldAlzheimerReport2011.pdf.

[26] Morley JE, Morris JC, Berg-Weger M (2015). Brain health: the importance of recognizing cognitive impairment: an IAGG consensus conference. J Am Med Dir Assoc.

[27] Liew TM (2020). Active case finding of dementia in ambulatory care settings: a comparison of three strategies. Eur J Neurol.

[28] Hendry K, Hill E, Quinn TJ, Evans J, Stott DJ (2015). Single screening questions for cognitive impairment in older people: a systematic review. Age Ageing.

[29] World Health Organization (2019). Integrated Care for Older People (ICOPE): Guidance for Person-Centred Assessment and Pathways in Primary Care.

[30] Galvin JE, Roe CM, Powlishta KK (2005). The AD8: a brief informant interview to detect dementia. Neurology (ECronicon).

[31] Pfeffer RI, Kurosaki TT, Harrah CH, Chance JM, Filos S (1982). Measurement of functional activities in older adults in the community. J Gerontol.

[32] Borson S, Scanlan J, Brush M, Vitaliano P, Dokmak A (2000). The mini‐cog: a cognitive “vital signs” measure for dementia screening in multi‐lingual elderly. Int J Geriat Psychiatry.

[33] Liew TM (2019). The optimal short version of montreal cognitive assessment in diagnosing mild cognitive impairment and dementia. J Am Med Dir Assoc.

[34] McDicken JA, Elliott E, Blayney G (2019). Accuracy of the short‐form montreal cognitive assessment: systematic review and validation. Int J Geriat Psychiatry.

[35] Livingston G, Huntley J, Liu KY (2024). Dementia prevention, intervention, and care: 2024 report of the Lancet Standing Commission. The Lancet.

[36] Fayers PM, Machin D (2007). Quality of Life: The Assessment, Analysis and Interpretation of Patient-Reported Outcomes.

[37] Tanwani R, Danquah MO, Butris N (2023). Diagnostic accuracy of ascertain dementia 8-item questionnaire by participant and informant-a systematic review and meta-analysis. PLoS ONE.

[38] Taylor-Rowan M, Nafisi S, Owen R (2023). Informant-based screening tools for dementia: an overview of systematic reviews. Psychol Med.

[39] (2017). A 4-step process to detecting cognitive impairment and earlier diagnosis of dementia: approaches and tools for primary care providers. https://issuu.com/gsastrategicalliances/docs/gsa_kaer_toolkit_4-step_process_to_detecting_cogni?fr=xKAE9_zU1NQ.

[40] Cordell CB, Borson S, Boustani M (2013). Alzheimer’s Association recommendations for operationalizing the detection of cognitive impairment during the Medicare Annual Wellness Visit in a primary care setting. Alzheimers Dement.

[41] Atri A, Dickerson BC, Clevenger C (2025). The Alzheimer’s association clinical practice guideline for the diagnostic evaluation, testing, counseling, and disclosure of suspected Alzheimer’s disease and related disorders (DETeCD-ADRD): validated clinical assessment instruments. Alzheimers Dement.

[42] Maslow K, Fortinsky RH (2018). Nonphysician care providers can help to increase detection of cognitive impairment and encourage diagnostic evaluation for dementia in community and residential care settings. Gerontologist.

[43] (2024). The World Bank in Singapore. World Bank Group.

[44] Liew TM, Foo JYH, Yang H (2025). PENSIEVE-AI a brief cognitive test to detect cognitive impairment across diverse literacy. Nat Commun.

[45] Patnode CD, Perdue LA, Rossom RC (2020). Screening for cognitive impairment in older adults: updated evidence report and systematic review for the US Preventive Services Task Force. JAMA.

[46] (2013). Diagnostic and Statistical Manual of Mental Disorders: DSM-5.

[47] Petersen RC, Morris JC (2005). Mild cognitive impairment as a clinical entity and treatment target. Arch Neurol.

[48] Kan CN, Zhang L, Cheng CY (2019). The informant AD8 can discriminate patients with dementia from healthy control participants in an Asian older cohort. J Am Med Dir Assoc.

[49] Jessen F, Wiese B, Bachmann C (2010). Prediction of dementia by subjective memory impairment: effects of severity and temporal association with cognitive impairment. Arch Gen Psychiatry.

[50] van Harten AC, Mielke MM, Swenson-Dravis DM (2018). Subjective cognitive decline and risk of MCI: the Mayo Clinic Study of Aging. Neurology (ECronicon).

[51] Jessen F, Amariglio RE, Buckley RF (2020). The characterisation of subjective cognitive decline. Lancet Neurol.

[52] Liew TM (2019). Depression, subjective cognitive decline, and the risk of neurocognitive disorders. Alzheimers Res Ther.

[53] Liew TM (2020). Subjective cognitive decline, anxiety symptoms, and the risk of mild cognitive impairment and dementia. Alzheimers Res Ther.

[54] Liew TM (2020). Trajectories of subjective cognitive decline, and the risk of mild cognitive impairment and dementia. Alzheimers Res Ther.

[55] Liew TM (2022). Subjective cognitive decline, APOE e4 allele, and the risk of neurocognitive disorders: age- and sex-stratified cohort study. Aust N Z J Psychiatry.

[56] Ng TP, Niti M, Chiam PC, Kua EH (2006). Physical and cognitive domains of the instrumental activities of daily living: validation in a multiethnic population of Asian older adults. J Gerontol A Biol Sci Med Sci.

[57] (2010). Bestglm: best subset GLM. https://cran.r-project.org/package=bestglm.

[58] Liew TM, Yap P (2020). A brief, 6-item scale for caregiver grief in dementia caregiving. Gerontologist.

[59] Liew TM, Yap P (2019). A 3-item screening scale for caregiver burden in dementia caregiving: scale development and score mapping to the 22-item Zarit burden interview. J Am Med Dir Assoc.

[60] Hosmer DW, Lemeshow S, Sturdivant RX (2013). Applied Logistic Regression.

[61] DeLong ER, DeLong DM, Clarke-Pearson DL (1988). Comparing the areas under two or more correlated receiver operating characteristic curves: a nonparametric approach. Biometrics.

[62] Yu J, Yap P, Liew TM (2019). The optimal short version of the Zarit Burden Interview for dementia caregivers: diagnostic utility and externally validated cutoffs. Aging Ment Health.

[63] Dautzenberg GMFG, Lijmer JG, Beekman ATF (2022). The Montreal Cognitive Assessment (MoCA) with a double threshold: improving the MoCA for triaging patients in need of a neuropsychological assessment. Int Psychogeriatr.

[64] Landsheer JA (2020). Impact of the prevalence of cognitive impairment on the accuracy of the Montreal Cognitive Assessment: the advantage of using two MoCA thresholds to identify error-prone test scores. Alzheimer Dis Assoc Disord.

[65] Thomann AE, Berres M, Goettel N, Steiner LA, Monsch AU (2020). Enhanced diagnostic accuracy for neurocognitive disorders: a revised cut-off approach for the Montreal Cognitive Assessment. Alz Res Therapy.

[66] Swartz RH, Cayley ML, Lanctôt KL (2016). Validating a pragmatic approach to cognitive screening in stroke prevention clinics using the Montreal Cognitive Assessment. Stroke.

[67] Jack CR, Andrews JS, Beach TG (2024). Revised criteria for diagnosis and staging of Alzheimer’s disease: Alzheimer’s Association Workgroup. Alzheimer’s Dementia.

[68] Bai W, Chen P, Cai H (2022). Worldwide prevalence of mild cognitive impairment among community dwellers aged 50 years and older: a meta-analysis and systematic review of epidemiology studies. Age Ageing.

[69] Hu C, Yu D, Sun X, Zhang M, Wang L, Qin H (2017). The prevalence and progression of mild cognitive impairment among clinic and community populations: a systematic review and meta-analysis. Int Psychogeriatr.

[70] Song WX, Wu WW, Zhao YY (2023). Evidence from a meta-analysis and systematic review reveals the global prevalence of mild cognitive impairment. Front Aging Neurosci.

[71] Prince M, Bryce R, Albanese E, Wimo A, Ribeiro W, Ferri CP (2013). The global prevalence of dementia: a systematic review and metaanalysis. Alzheimer's Dementia.

[72] Fiest KM, Jetté N, Roberts JI (2016). The prevalence and incidence of dementia: a systematic review and meta-analysis. Can J Neurol Sci.

[73] Cao Q, Tan CC, Xu W (2020). The prevalence of dementia: a systematic review and meta-analysis. J Alzheimers Dis.

[74] Hanley JA, McNeil BJ (1983). A method of comparing the areas under receiver operating characteristic curves derived from the same cases. Radiology.

[75] Mental Capacity Act 2008. Singapore Statutes Online.

[76] Informant questionnaire for cognitive impairment. https://brainhealth-iq2plus.hf.space/.

[77] Cai Y, Qiu P, Wan Y (2021). Establishing cut-off scores for the self-rating AD8 based on education level. Geriatr Nurs (Lond).

[78] Chen HH, Sun FJ, Yeh TL (2018). The diagnostic accuracy of the Ascertain Dementia 8 questionnaire for detecting cognitive impairment in primary care in the community, clinics and hospitals: a systematic review and meta-analysis. Fam Pract.

[79] Barthold D, Marcum ZA, Chen S (2021). Difficulty with taking medications is associated with future diagnosis of Alzheimer’s disease and related dementias. J Gen Intern Med.

[80] (2024). Take the type 2 risk test. American Diabetes Association.

[81] American Diabetes Association Professional Practice Committee (2024). 2. Diagnosis and Classification of Diabetes: Standards of Care in Diabetes-2024. Diabetes Care.

[82] Kalaria R, Maestre G, Mahinrad S (2024). The 2022 symposium on dementia and brain aging in low- and middle-income countries: Highlights on research, diagnosis, care, and impact. Alzheimers Dement.

[83] Jacova C, Kertesz A, Blair M, Fisk JD, Feldman HH (2007). Neuropsychological testing and assessment for dementia. Alzheimer's Dementia.

[84] Ang LC, Yap P, Tay SY, Koay WI, Liew TM (2023). Examining the validity and utility of Montreal Cognitive Assessment domain scores for early neurocognitive disorders. J Am Med Dir Assoc.

