# Supplementary material for: An Ultra-Brief Informant Questionnaire for Case Finding of Cognitive Impairment Across Diverse Literacy: Diagnostic Accuracy Study
Source: JMIR Aging. 2026 Jan 6;9:e72963. doi: 10.2196/72963 (PMC12772941; doi:10.2196/72963)
Supplement: Multimedia Appendix 1 [file aging-v9-e72963-s001.pdf]

MULTIMEDIA APPENDIX

Supplementary Method S1. Samples of the study's publicity materials.

(A) <u>Study Banner (for community roadshows)</u>

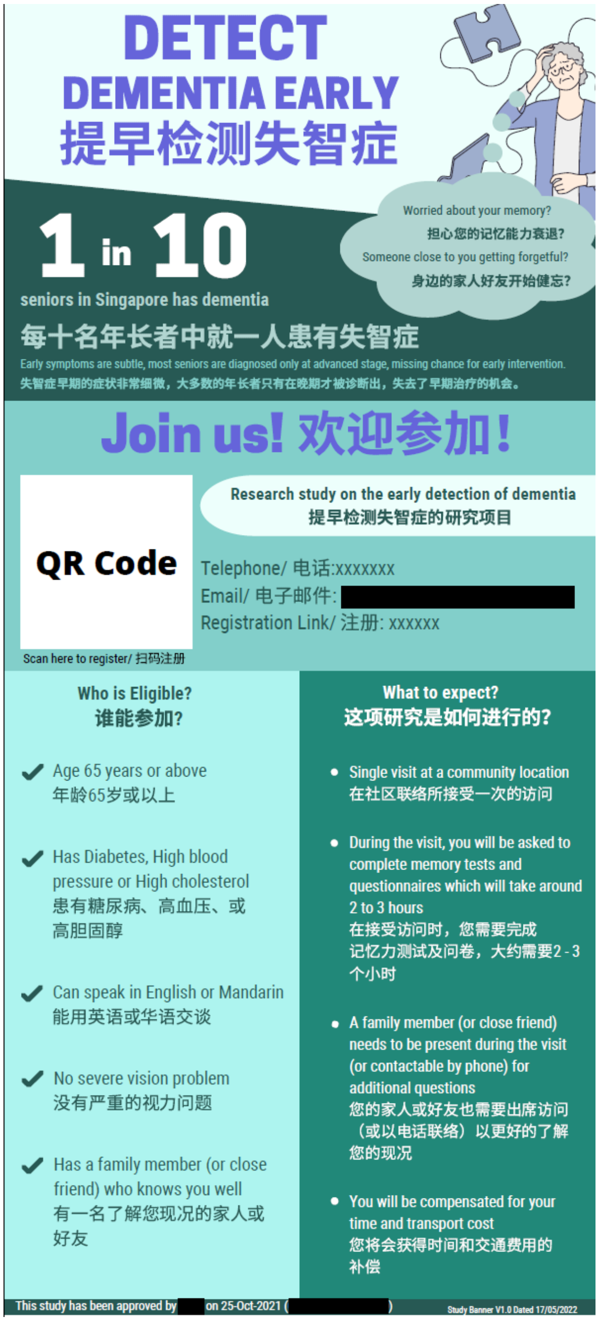

(B) Study Poster (English and Chinese versions)

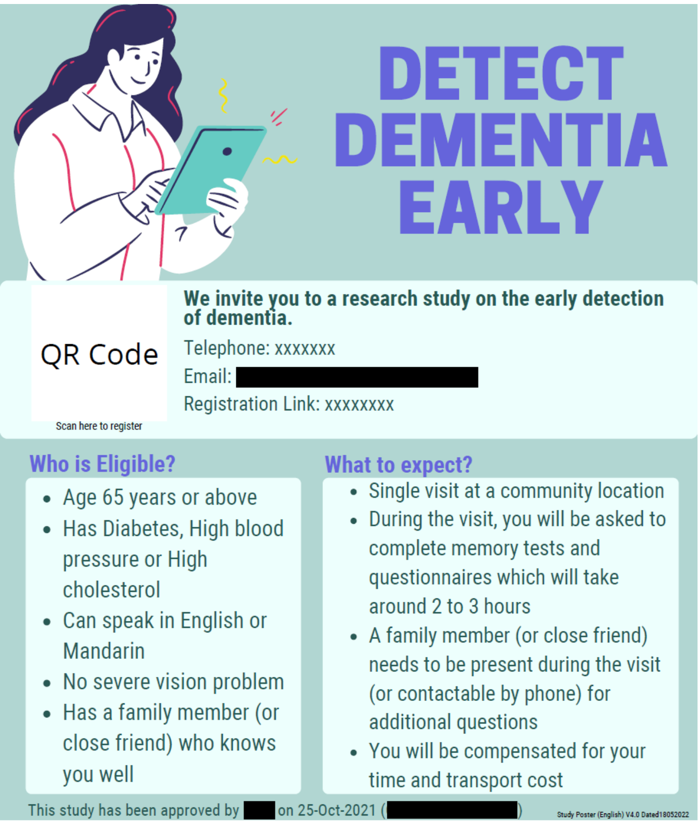

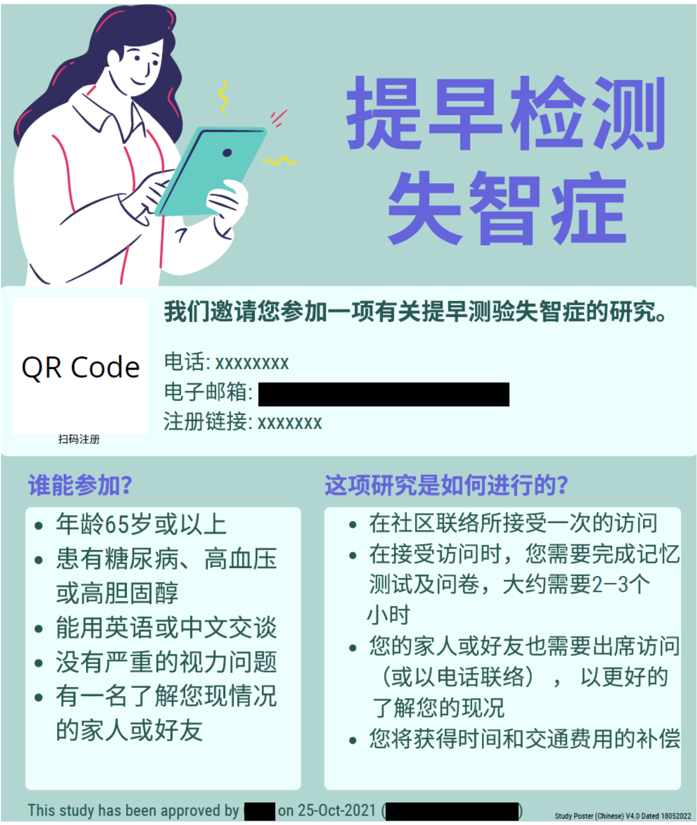

(C) Study Brochure (English and Chinese versions)

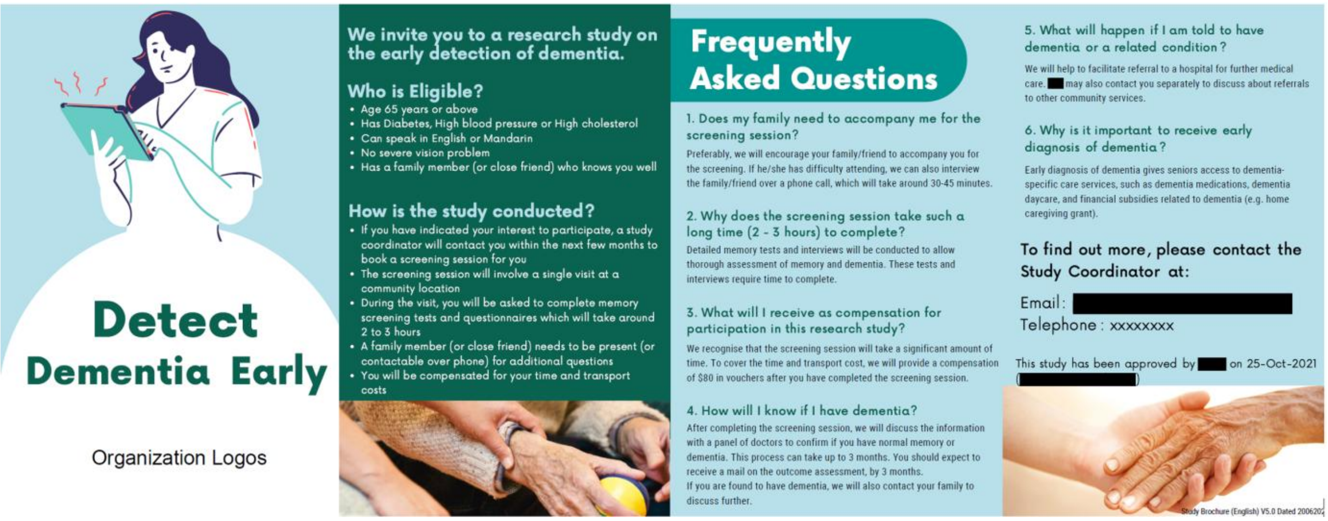

Detect Dementia Early

Organization Logos

We invite you to a research study on the early detection of dementia.

Who is Eligible?

- Age 65 years or above
- Has Diabetes, High blood pressure or High cholesterol
- Can speak in English or Mandarin
- No severe vision problem
- Has a family member (or close friend) who knows you well

How is the study conducted?

- If you have indicated your interest to participate, a study coordinator will contact you within the next few months to book a screening session for you
- The screening session will involve a single visit at a community location
- During the visit, you will be asked to complete memory screening tests and questionnaires which will take around 2 to 3 hours
- A family member (or close friend) needs to be present (or contactable over phone) for additional questions
- You will be compensated for your time and transport costs

Frequently Asked Questions

1. Does my family need to accompany me for the screening session?

Preferably, we will encourage your family/friend to accompany you for the screening. If he/she has difficulty attending, we can also interview the family/friend over a phone call, which will take around 30-45 minutes.

2. Why does the screening session take such a long time (2 - 3 hours) to complete?

Detailed memory tests and interviews will be conducted to allow thorough assessment of memory and dementia. These tests and interviews require time to complete.

3. What will I receive as compensation for participation in this research study?

We recognise that the screening session will take a significant amount of time. To cover the time and transport cost, we will provide a compensation of $80 in vouchers after you have completed the screening session.

4. How will I know if I have dementia?

After completing the screening session, we will discuss the information with a panel of doctors to confirm if you have normal memory or dementia. This process can take up to 3 months. You should expect to receive a mail on the outcome assessment, by 3 months.
If you are found to have dementia, we will also contact your family to discuss further.

5. What will happen if I am told to have dementia or a related condition?

We will help to facilitate referral to a hospital for further medical care. [redacted] may also contact you separately to discuss about referrals to other community services.

6. Why is it important to receive early diagnosis of dementia?

Early diagnosis of dementia gives seniors access to dementia-specific care services, such as dementia medications, dementia daycare, and financial subsidies related to dementia (e.g. home caregiving grant).

To find out more, please contact the Study Coordinator at:

Email: [redacted]
Telephone : xxxxxxxx

This study has been approved by [redacted] on 25-Oct-2021 ([redacted])

Study Brochure (English) V5.0 Dated 2006202[illegible]

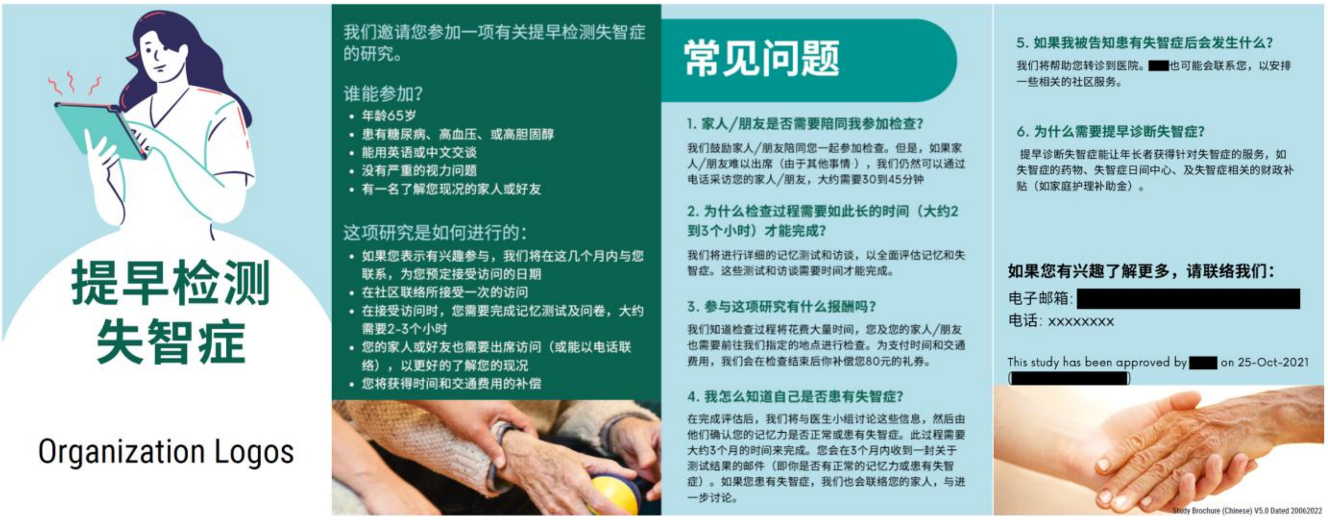

提早检测失智症

Organization Logos

我们邀请您参加一项有关提早检测失智症的研究。

谁能参加?

- 年龄65岁
- 患有糖尿病、高血压、或高胆固醇
- 能用英语或中文交谈
- 没有严重的视力问题
- 有一名了解您现况的家人或好友

这项研究是如何进行的:

- 如果您表示有兴趣参与，我们将在这几个月内与您联系，为您预定接受访问的日期
- 在社区联络所接受一次的访问
- 在接受访问时，您需要完成记忆测试及问卷，大约需要2-3个小时
- 您的家人或好友也需要出席访问（或能以电话联络），以更好的了解您的现况
- 您将获得时间和交通费用的补偿

常见问题

1. 家人/朋友是否需要陪同我参加检查?

我们鼓励家人/朋友陪同您一起参加检查。但是，如果家人/朋友难以出席（由于其他事情），我们仍然可以通过电话采访您的家人/朋友，大约需要30到45分钟

2. 为什么检查过程需要如此长的时间（大约2到3个小时）才能完成?

我们将进行详细的记忆测试和访谈，以全面评估记忆和失智症。这些测试和访谈需要时间才能完成。

3. 参与这项研究有什么报酬吗?

我们知道检查过程将花费大量时间，您及您的家人/朋友也需要前往我们指定的地点进行检查。为支付时间和交通费用，我们会在检查结束后你补偿您80元的礼券。

4. 我怎么知道自己是否患有失智症?

在完成评估后，我们将与医生小组讨论这些信息，然后由他们确认您的记忆力是否正常或患有失智症。此过程需要大约3个月的时间来完成。您会在3个月内收到一封关于测试结果的邮件（即你是否有正常的记忆力或患有失智症）。如果您患有失智症，我们也会联络您的家人，与进一步讨论。

5. 如果我被告知患有失智症后会发生什么?

我们将帮助您转诊到医院。[redacted]也可能会联系您，以安排一些相关的社区服务。

6. 为什么需要提早诊断失智症?

提早诊断失智症能让年长者获得针对失智症的服务，如失智症的药物、失智症日间中心、及失智症相关的财政补贴（如家庭护理补助金）。

如果您有兴趣了解更多，请联络我们:

电子邮箱: [redacted]
电话: xxxxxxxx

This study has been approved by [redacted] on 25-Oct-2021 ([redacted])

Study Brochure (Chinese) V5.0 Dated 20062022

Supplementary Method S2. Descriptions of the comprehensive assessments for cognitive impairment.

Comprehensive assessments of the participants were conducted by trained research coordinators, and included semi-structured interviews with participants and their informants, detailed neuropsychological testing, and observational notes of participants' behavior during assessments. The assessments were conducted in either English or Chinese Mandarin, taking into account the language preference of participants and their informants. The assessment protocol is described in the ensuing paragraphs, while full details of the assessment tools are further described in **Supplementary Method S3**.

In the semi-structured interview with informants, the research coordinator first administered questionnaires to identify informant's concerns related to: (1) subjective cognitive difficulties (Everyday Cognition scale–12 item version, ECog-12);[1] (2) instrumental activities of daily living (modified Lawton scale);[2] (3) basic activities of daily living (Katz scale);[3] and (4) neuropsychiatric symptoms (Neuropsychiatric Inventory–Questionnaire, NPI-Q).[4] For items that were answered affirmatively in each questionnaire, the research coordinator then conducted semi-structured interviews to further clarify with informants on the nature of the symptoms, focusing on: (a) current descriptions and examples of the symptoms; (b) frequency of symptoms; and (c) onset and evolution of symptoms over time.

Similar semi-structured interviews were conducted with the participants, focusing primarily on subjective cognitive difficulties as identified by participants (ECog-12).[1] In addition, participants were also administered a depression scale (Patient Health Questionnaire 9-item depression scale, PHQ-9)[5] and an anxiety scale (Generalized Anxiety Disorder 7-item scale, GAD-7),[6] to identify those with clinically-significant depression (based on PHQ-9's diagnostic algorithm for major depression)[5] or clinically-significant anxiety (based on GAD≥10).[6]

Participants were then administered a global cognitive test (Montreal Cognitive Assessment, MoCA)[7] that measures six domains (namely Visuospatial abilities, Memory, Language, Attention, Executive function and Orientation),[8] as well as a brief neuropsychological battery that covered the domains of Visuospatial abilities (*Benson Complex Figure Copy*), Working memory (*Craft Story 21 Immediate Recall*), Delayed memory (*Craft Story 21 Delayed Recall* and *Benson Complex Figure Recall*), Language (*Verbal Fluency–Animal*), Attention/Processing speed (*Trail Making Test–Part A*), and Executive function (*Trail Making Test–Part B*).[9] After the assessments, the research coordinators took down observation notes on participants' behaviors throughout the sessions; as well as completed a brief delirium scale (Short Confusion Assessment Method, Short CAM),[10] along with additional observational notes for each item that was answered affirmatively in Short CAM.

On a monthly basis, consensus conference was conducted to determine the clinical diagnoses (i.e. dementia, MCI, or normal cognition) and Clinical Dementia Rating scoring (CDR) of the recruited participants, based on responses from the comprehensive assessment. The consensus conference involved a panel of three dementia specialists, comprising a geriatric psychiatrist (TML) and two other

co-investigators who could be a geriatrician (KFY), neurologists (SKST, KN, WL) or psychologists (SYT, WIK).

To ensure the reliability and integrity of the research assessments, a quality assurance protocol was implemented throughout the study period. Prior to initial training of the research protocol, all research coordinators completed several sessions of observations of clinical and neuropsychological assessments in routine memory clinics. Thereafter, the research coordinators received detailed briefings on the assessment tools (by TML, SYT and WIK), followed by multiple trials of role-playing to ensure their familiarity with the tools before conducting the first actual assessment. The research coordinators then received direct supervision from TML when they were conducting the initial assessments. On a monthly basis, all the assessments were audited by the study co-investigators, with regular feedback given to the research coordinators on potential areas of improvement. Every 6 months, all the collected data were randomly audited by the principal investigator (TML) to ensure quality and accuracy of the data.

Supplementary Method S3. Further descriptions of the assessment tools that were used in the study.

ECog-12[1] is a brief questionnaire that measures cognitively-relevant everyday abilities. It can be completed by self or informant, and comprises 12 items that cover six cognitively-relevant domains: Everyday Memory, Everyday Language, Everyday Visuospatial Abilities, Everyday Planning, Everyday Organization, and Everyday Divided Attention. For each item, the respondent compared the current level of everyday abilities with those of 10 years earlier. In this way, each individual served as his own control. Ratings were made on a 4-point scale: 1=better or no change compared with 10 years earlier, 2=questionable/occasionally worse, 3=consistently a little worse, 4=consistently much worse. ECog-12 was developed using item response theory to cover a wide range of everyday ability.[1] In the original validation study,[1] it strongly correlated with established functional measures and neuropsychological scores, only weakly with age and education, and demonstrated high internal consistency. It also showed excellent discrimination between those with and without cognitive impairment. In a recent population-based cohort study, ECog-12 was even shown to be a useful measure to predict mild cognitive impairment among individuals with normal cognition at baseline,[11] which provides further evidence on its construct validity as a measure of cognitively-relevant everyday abilities.

Modified Lawton scale[2] is an informant-based questionnaire that assesses participant's current level of dependence in performing eight instrumental activities of daily living (using the telephone, getting about, grocery shopping, preparing meals, doing housework, doing laundry, taking medicines, and managing money). For each item, the informant indicated whether the participant: 0=were unable to do at all, 1=needed some assistance, or 2=needed no help. Only those receiving assistance from a person (as opposed to a device) were considered to be receiving help. Modified Lawton scale has been shown to have good validity and reliability in Singapore.[2]

Katz scale[3] is an informant-based questionnaire that assesses participant's current level of dependence in performing six basic activities of daily living (bathing, dressing, toileting, transferring, continence, and feeding). For each item, the informant indicated whether the participant: 0=were unable to do at all, 1=needed some assistance, or 2=needed no help. Only those receiving assistance from a person (as opposed to a device) were considered to be receiving help.

Neuropsychiatric Inventory–Questionnaire (NPI-Q)[4] was included as part of the standardized assessments to screen for a wide range of psychiatric-related conditions that may mimic dementia. NPI-Q is an informant-based questionnaire that screens for the presence of neuropsychiatric symptoms in the past month. It has 12 items that assess neuropsychiatric symptoms in 12 domains, namely depression, anxiety, apathy, sleep, appetite, agitation, irritability, disinhibition, elation, motor disturbance, delusions, and hallucinations. Each of its item was rated by informants on a 4-point Likert scale: 0=Not present, 1=Mild (noticeable, but not a significant change), 2=Moderate (significant, but not a dramatic change), and 3=Severe (very marked or prominent; a dramatic change). Although NPI-Q was originally designed for use among patients with cognitive impairment,[12] it has also been shown to be useful in capturing

clinically-significant neuropsychiatric symptoms even among those with normal cognition,[13] and is widely-used among those with normal cognition in Alzheimer's Research Centers across USA.[13] NPI-Q has previously been validated in Singapore.[14]

Patient Health Questionnaire 9-item depression scale (PHQ-9)[5] and **Generalized Anxiety Disorder 7-item scale (GAD-7)**[6] are among the best validated and most commonly used depression and anxiety measures, respectively.[15] PHQ-9 consists of 9 items representing the criterion symptoms for DSM-5 (Diagnostic and Statistical Manual of Mental Disorders–Fifth Edition) major depressive disorder.[5] Participants were asked how much each symptom has bothered them over the past 2 weeks, with response options of: 0=not at all, 1=several days, 2=more than half the days, and 3=nearly every day. PHQ-9 has an established diagnostic algorithm[5] that maps its item responses to DSM-5 criteria to identify those with clinically-significant depression. GAD-7 has 7 items with response options identical to the PHQ-9 and therefore can be scored as a continuous variable from 0 to 21, with higher scores representing more severe anxiety and scores≥10 indicating clinically-significant anxiety.[6] Although originally developed as a measure to detect generalized anxiety disorder,[6] the operating characteristics of the GAD-7 are nearly as good for the other common anxiety disorders in clinical practice, such as panic disorder, social anxiety disorder, and posttraumatic stress disorder.[16] PHQ-9 and GAD-7 had been shown to have strong internal and test-retest reliability as well as construct and factor-structure validity.[17]

Montreal Cognitive Assessment (MoCA)[7] is a widely used global cognitive measure. It comprises 12 individual tests which evaluate the cognitive performance across six different domains, namely Visuospatial abilities, Memory, Language, Attention, Executive function and Orientation.[8] The test is interviewer-administered and has a maximum score of 30 with higher scores corresponding to better cognition. Compared to the traditionally popular MMSE, MoCA includes more robust measures of visuospatial and executive function, and has been shown to have better utility in detecting early cognitive deficits.[7,18,19] The local version of MoCA was be used in this study.[20]

The brief neuropsychological battery included seven neuropsychological tests which cover the domains of Visuospatial abilities (*Benson Complex Figure Copy*), Working memory (*Craft Story 21 Immediate Recall*), Delayed memory (*Craft Story 21 Delayed Recall* and *Benson Complex Figure Recall*), Language (*Verbal Fluency–Animal*), Attention/Processing speed (*Trail Making Test–Part A*), and Executive function (*Trail Making Test–Part B*).[9] These seven neuropsychological tests were based on a subset of the original, Alzheimer's disease Research Centers' Neuropsychological Test Battery (Version 3) (ADRC-NTB3), which is among the most widely-used standardized battery for evaluation of cognitive impairment.[9] The seven tests were selected from the original ADRC-NTB3 based on the principles: (1) the brief neuropsychological battery should include at least one test from each cognitive domains, while ensuring the total administration time can be kept within an average of 60 minutes (to accommodate study feasibility); and (2) the included tests should have minimal language bias (i.e. we excluded tests that relied heavily on language abilities, e.g. Phonemic Fluency and Picture Naming). The seven neuropsychological tests are briefly described here, with the full details available in a original

publication.[9] *Craft Story 21* assesses the ability to provide verbatim recall of a short story immediately after hearing it, and 20 minutes later.[21] *Benson Complex Figure Copy* assesses the ability to copy a simplified form of the Rey-Osterrieth figure, and to draw from memory the same figure after 20 minutes.[22] *Verbal Fluency–Animal* measures the number of animals that a participant can name in 1 minute. *Trail Making Test (Part A)* requires the participants to connect the circles in ascending numerical order (from 1 to 25) as quickly as possible, while *Trail Making Test (Part B)* requires the participants to connect the circles while alternating between numbers and letters in an ascending order. *Trail Making Test (Part A and Part B)* are measured by the correct lines connected divided by the time to completion, as this computation was previously shown to provide more accurate Z-scores.[9] The Z-scores for the seven neuropsychological tests were computed using published age-, sex- and education-adjusted normative calculator;[9] while the global Z-score was computed by averaging the Z-scores of the seven neuropsychological tests.

Short Confusion Assessment Method (Short CAM)[10] is a brief instrument that enables non-psychiatrically-trained clinicians to identify delirium quickly and accurately in both clinical and research settings.[10] It is usually administered by interviewers based on their observations of the participants during a 10–20 minute interview. The key features that are captured in Short CAM include: (1) acute change or fluctuation (any symptom); (2) inattention; (3) disorganized thinking; and (4) altered level of consciousness. Short CAM is among the most widely-used delirium scale, and it has been shown in the literature to have good validity and reliability for detecting delirium.[10]

Clinical Dementia Rating (CDR) (*CDR® Dementia Staging Instrument*)[23] is a well-validated and widely-used scale for staging of cognitive impairment.[24] CDR requires interviews with both participant and informant to rate performance in 6 domains (memory, orientation, judgment and problem solving, community affairs, home and hobbies, and personal care). Based on published rules,[23] responses from the 6 domains are used to assign a global CDR score to indicate the severity of cognitive impairment: 0=normal cognition, 0.5=questionable cognitive impairment, 1=mild dementia, 2=moderate dementia, and 3=severe dementia.

Supplementary Method S4. Further descriptions of the post hoc power analyses.

To assess the statistical adequacy of the sample size for detecting meaningful diagnostic performance, we performed post hoc power analyses using PASS Sample Size Software (version 15.0.5), applying the formula described by Hanley and McNeil.[25] Analyses were based on a two-sided z-test at a significance level of 0.05, with a null hypothesis AUC of 75.0% (reflecting average, minimally-acceptable performance for a questionnaire), and alternative hypothesis AUCs based on observed results for the IQ2+.

Power calculations were conducted for the overall Test sample and stratified by education subgroups:

- **Overall Test sample (n = 577):** 76 participants with MCI/dementia and 481 with normal cognition provided 90% power to detect an observed AUC of 85.3% (difference from null = 10.3%).
- **≤10 years of education (n = 327):** 59 participants with MCI/dementia and 268 with normal cognition provided 72% power to detect an observed AUC of 84.5% (difference = 9.5%).
- **>10 years of education (n = 230):** 17 participants with MCI/dementia and 213 with normal cognition provided 19% power to detect an observed AUC of 83.3% (difference = 8.3%).

Overall, the Test sample provides robust power for detecting meaningful diagnostic performance of IQ2+. Power was sufficient in the lower-education subgroup, but limited in the higher-education subgroup due to fewer positive cases.

Figure S1. Model selection with fivefold cross-validation in the Training sample (n=1,299).

Note: Models with lower prediction errors are considered better; among these, the 12-item model had the lowest prediction error. However, based on the 'one-standard-error' rule, the four-item model (comprising iADL–Medications, Worry about cognition–Informant, Age, and Years of education) was selected to constitute the new ultra-brief questionnaire, as it was the most parsimonious model that still fell within one standard error of the model with the lowest prediction error (indicated by the two horizontal dotted lines in the plot). The 'one-standard error' rule is an established method to avoid selecting an overfitted model and to ensure replicability of findings in independent samples.[26]

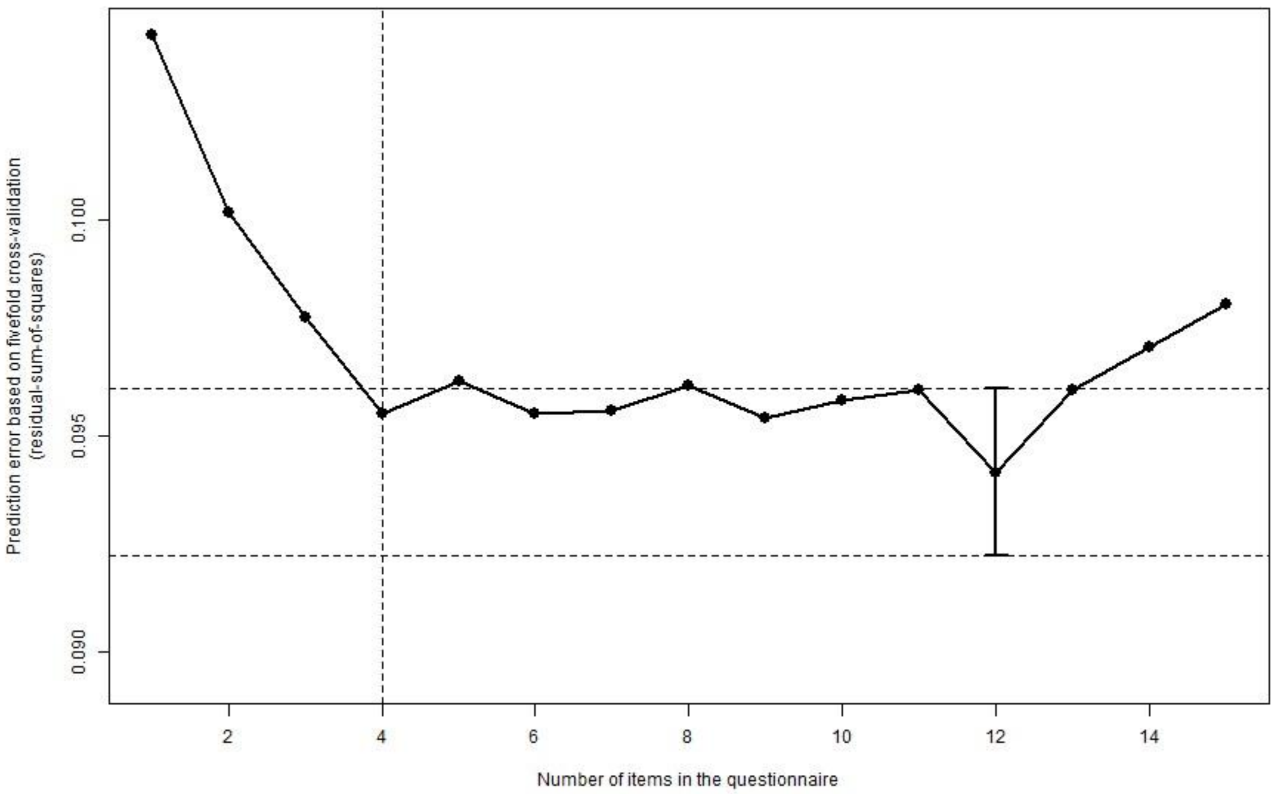

Table S1. Characteristics of the study informants (n=1,856)

Variable	Overall sample (n=1,856)
Age, median (IQR)	67 (52, 72)
Male sex, n (%)	655 (35.3)
Relationship to the participant, n (%)	
Spouse	897 (48.3)
Children/Children-in-law	506 (27.3)
Other family member or relative	255 (13.7)
Friend/Neighbour	167 (9.0)
Care worker/Domestic helper	31 (1.7)
Frequency of contact with participant, n (%)	
Daily	1,340 (72.2)
At least once a week	405 (21.8)
At least once a month	89 (4.8)
Once every few months or lesser	22 (1.2)

IQR, interquartile range.

Table S2. Comparison of participant characteristics across the Training and Test samples.

Variable	Overall sample (n=1,856)	Training sample (n=1,299)	Test sample (n=557)	P value [a]
Age, median (IQR)	72 (68, 76)	72 (68, 76)	71 (68, 75)	0.319
Years of education, median (IQR)	10 (9, 13)	10 (9, 13)	10 (9, 12)	0.193
Male sex, n (%)	688 (37.1)	487 (37.5)	201 (36.1)	0.566
Ethnicity, n (%)				0.407
Chinese	1,735 (93.5)	1,209 (93.1)	526 (94.4)	
Malay/Indian	96 (5.2)	73 (5.6)	23 (4.1)	
Eurasian/Caucasian/Others	25 (1.3)	17 (1.3)	8 (1.4)	
MoCA total score, median (IQR)	26 (24, 28)	26 (24, 28)	26 (24, 28)	0.514
NTB Global Z-scores, median (IQR)	-0.2 (-0.6, 0.1)	-0.2 (-0.6, 0.1)	-0.2 (-0.6, 0.2)	0.460
Global CDR, n (%)				0.143
0	1,570 (84.6)	1,094 (84.2)	476 (85.5)	
0.5	255 (13.7)	188 (14.5)	67 (12.0)	
1	22 (1.2)	13 (1.0)	9 (1.6)	
2	8 (0.4)	4 (0.3)	4 (0.7)	
3	1 (0.1)	0 (0.0)	1 (0.2)	
Diagnosis, n (%)				0.837
Normal cognition	1,601 (86.3)	1,120 (86.2)	481 (86.4)	
Mild cognitive impairment	207 (11.2)	147 (11.3)	60 (10.8)	
Dementia	48 (2.6)	32 (2.5)	16 (2.9)	

IQR, interquartile range; MoCA, Montreal Cognitive Assessment; NTB, neuropsychological battery; CDR, Clinical Dementia Rating.

[a] Test of difference between Training and Test samples: chi-square test for categorical variables, and Wilcoxon rank-sum test for continuous variables. Bold-faced p-values are <0.05.

Table S3. The test statistics of IQ2+ in identifying cognitive impairment in the Test sample.

Probability score (%)	MCI/Dementia versus Normal cognition				Dementia versus Non-dementia			
	Sensitivity (%)	Specificity (%)	NPV (%)	PPV (%)	Sensitivity (%)	Specificity (%)	NPV (%)	PPV (%)
≥4	100.0	7.5	100.0	14.6	100.0	6.7	100.0	3.1
≥5	98.7	26.8	99.2	17.6	100.0	24.0	100.0	3.7
≥6	90.8	43.7	96.8	20.3	100.0	40.1	100.0	4.7
≥7	88.2	55.9	96.8	24.0	100.0	51.4	100.0	5.7
≥8	88.2	63.6	97.1	27.7	100.0	58.2	100.0	6.6
≥9	86.8	67.6	97.0	29.7	100.0	61.9	100.0	7.2
≥10	84.2	70.7	96.6	31.2	100.0	65.1	100.0	7.8
≥11	82.9	72.3	96.4	32.1	100.0	66.7	100.0	8.2
**≥12** [a]	**80.3**	**74.8**	**96.0**	**33.5**	93.8	69.1	99.7	8.2
≥13	78.9	75.9	95.8	34.1	93.8	70.2	99.7	8.5
≥14	77.6	77.5	95.6	35.3	93.8	71.9	99.7	9.0
≥15	76.3	79.2	95.5	36.7	93.8	73.6	99.7	9.5
≥16	75.0	82.5	95.4	40.4	93.8	76.7	99.8	10.6
≥17	73.7	84.4	95.3	42.7	93.8	78.6	99.8	11.5
≥18	71.1	85.7	94.9	43.9	93.8	80.0	99.8	12.2
≥19	67.1	86.7	94.3	44.3	93.8	81.5	99.8	13.0
≥20	64.5	88.6	94.0	47.1	93.8	83.5	99.8	14.4
≥21	60.5	90.0	93.5	48.9	93.8	85.4	99.8	16.0
≥22	59.2	91.1	93.4	51.1	93.8	86.5	99.8	17.0
≥23	55.3	92.3	92.9	53.2	93.8	88.2	99.8	19.0
≥24	53.9	93.1	92.8	55.4	93.8	89.1	99.8	20.3
≥25	52.6	93.3	92.6	55.6	93.8	89.5	99.8	20.8
≥26	50.0	94.0	92.2	56.7	93.8	90.4	99.8	22.4
≥27	48.7	94.2	92.1	56.9	93.8	90.8	99.8	23.1
≥28	48.7	95.0	92.1	60.7	93.8	91.5	99.8	24.6
≥29	46.1	96.0	91.8	64.8	93.8	92.8	99.8	27.8
≥30	44.7	96.3	91.7	65.4	93.8	93.2	99.8	28.8
≥31	44.7	96.3	91.7	65.4	93.8	93.2	99.8	28.8
≥32	40.8	96.5	91.2	64.6	87.5	93.7	99.6	29.2
≥33	40.8	96.7	91.2	66.0	87.5	93.9	99.6	29.8
≥34	39.5	96.7	91.0	65.2	87.5	94.1	99.6	30.4
≥35	38.2	96.7	90.8	64.4	87.5	94.3	99.6	31.1
≥36	38.2	96.9	90.8	65.9	87.5	94.5	99.6	31.8
≥37	36.8	97.3	90.7	68.3	87.5	95.0	99.6	34.1
≥38	36.8	97.5	90.7	70.0	87.5	95.2	99.6	35.0
≥39	36.8	97.5	90.7	70.0	87.5	95.2	99.6	35.0
≥40	35.5	97.9	90.6	73.0	87.5	95.7	99.6	37.8
≥41	35.5	97.9	90.6	73.0	87.5	95.7	99.6	37.8
≥42	34.2	97.9	90.4	72.2	87.5	95.9	99.6	38.9
≥43	34.2	98.1	90.4	74.3	87.5	96.1	99.6	40.0
≥44	32.9	98.1	90.2	73.5	87.5	96.3	99.6	41.2

≥45	32.9	98.1	90.2	73.5	87.5	96.3	99.6	41.2
≥46	32.9	98.3	90.3	75.8	87.5	96.5	99.6	42.4
≥47	30.3	98.3	89.9	74.2	87.5	96.9	99.6	45.2
≥48	28.9	98.3	89.8	73.3	87.5	97.0	99.6	46.7
≥49	28.9	98.5	89.8	75.9	87.5	97.2	99.6	48.3
≥50	28.9	98.5	89.8	75.9	87.5	97.2	99.6	48.3
≥51	27.6	98.8	89.6	77.8	87.5	97.6	99.6	51.9
**≥52** [a]	**27.6**	**99.0**	**89.6**	**80.8**	87.5	97.8	99.6	53.8
≥53	27.6	99.0	89.6	80.8	87.5	97.8	99.6	53.8
≥54	27.6	99.0	89.6	80.8	87.5	97.8	99.6	53.8
≥55	26.3	99.0	89.5	80.0	87.5	98.0	99.6	56.0
≥56	26.3	99.0	89.5	80.0	87.5	98.0	99.6	56.0
≥57	26.3	99.0	89.5	80.0	87.5	98.0	99.6	56.0
≥58	26.3	99.2	89.5	83.3	87.5	98.2	99.6	58.3
≥59	25.0	99.2	89.3	82.6	81.3	98.2	99.4	56.5
≥60	23.7	99.2	89.2	81.8	75.0	98.2	99.3	54.5
≥61	23.7	99.2	89.2	81.8	75.0	98.2	99.3	54.5
≥62	23.7	99.2	89.2	81.8	75.0	98.2	99.3	54.5
≥63	23.7	99.2	89.2	81.8	75.0	98.2	99.3	54.5
≥64	23.7	99.2	89.2	81.8	75.0	98.2	99.3	54.5
≥65	23.7	99.2	89.2	81.8	75.0	98.2	99.3	54.5
≥66	23.7	99.2	89.2	81.8	75.0	98.2	99.3	54.5
≥67	23.7	99.2	89.2	81.8	75.0	98.2	99.3	54.5
≥68	23.7	99.2	89.2	81.8	75.0	98.2	99.3	54.5
≥69	23.7	99.2	89.2	81.8	75.0	98.2	99.3	54.5
≥70	23.7	99.4	89.2	85.7	75.0	98.3	99.3	57.1
≥71	22.4	99.4	89.0	85.0	75.0	98.5	99.3	60.0
≥72	19.7	99.4	88.7	83.3	62.5	98.5	98.9	55.6
≥73	18.4	99.4	88.5	82.4	56.3	98.5	98.7	52.9
≥74	17.1	99.4	88.4	81.3	50.0	98.5	98.5	50.0
≥75	14.5	99.4	88.0	78.6	43.8	98.7	98.3	50.0
≥76	14.5	99.4	88.0	78.6	43.8	98.7	98.3	50.0
≥77	14.5	99.4	88.0	78.6	43.8	98.7	98.3	50.0
≥78	13.2	99.4	87.9	76.9	43.8	98.9	98.3	53.8
≥79	11.8	99.6	87.7	81.8	37.5	99.1	98.2	54.5
≥80	11.8	99.6	87.7	81.8	37.5	99.1	98.2	54.5
≥81	11.8	99.6	87.7	81.8	37.5	99.1	98.2	54.5
≥82	9.2	99.6	87.4	77.8	37.5	99.4	98.2	66.7
≥83	9.2	99.6	87.4	77.8	37.5	99.4	98.2	66.7
≥84	6.6	99.6	87.1	71.4	25.0	99.4	97.8	57.1
≥85	6.6	99.6	87.1	71.4	25.0	99.4	97.8	57.1
≥86	6.6	99.6	87.1	71.4	25.0	99.4	97.8	57.1
≥87	3.9	99.8	86.8	75.0	12.5	99.6	97.5	50.0
≥88	3.9	100.0	86.8	100.0	12.5	99.8	97.5	66.7

≥89	3.9	100.0	86.8	100.0	12.5	99.8	97.5	66.7
≥90	3.9	100.0	86.8	100.0	12.5	99.8	97.5	66.7
≥91	3.9	100.0	86.8	100.0	12.5	99.8	97.5	66.7
≥92	2.6	100.0	86.7	100.0	6.3	99.8	97.3	50.0
≥93	2.6	100.0	86.7	100.0	6.3	99.8	97.3	50.0
≥94	2.6	100.0	86.7	100.0	6.3	99.8	97.3	50.0

IQ2+, the Informant Questionnaire for cognitive impairment–Two items (plus demographics); MCI, mild cognitive impairment; NPV, negative predictive value; PPV, positive predictive value.

[a] Bold-faced values indicate the two optimal cutoffs (i.e. the lower cutoff has high sensitivity and NPV, while the upper cutoff has high specificity and PPV).

Table S4. The test statistics of IQ2+ in identifying cognitive impairment in the Test sample, with the prevalence of MCI/Dementia artificially adjusted to 20%.[a]

Probability score (%)	MCI/Dementia versus Normal cognition				Dementia versus Non-dementia			
	Sensitivity (%)	Specificity (%)	NPV (%)	PPV (%)	Sensitivity (%)	Specificity (%)	NPV (%)	PPV (%)
≥4	100.0	7.0	100.0	21.2	100.0	5.9	100.0	5.3
≥5	98.4	25.0	98.5	24.7	100.0	21.4	100.0	6.3
≥6	92.2	40.2	95.4	27.8	100.0	35.5	100.0	7.5
≥7	89.1	53.9	95.2	32.6	100.0	47.7	100.0	9.1
≥8	89.1	62.1	95.8	37.0	100.0	54.6	100.0	10.4
≥9	87.5	66.0	95.5	39.2	100.0	58.2	100.0	11.2
≥10	84.4	68.4	94.6	40.0	100.0	60.9	100.0	11.9
≥11	84.4	69.9	94.7	41.2	100.0	62.2	100.0	12.2
**≥12** [b]	**81.3**	**73.4**	**94.0**	**43.3**	93.8	65.5	99.5	12.5
≥13	79.7	74.2	93.6	43.6	93.8	66.4	99.5	12.8
≥14	78.1	76.2	93.3	45.0	93.8	68.4	99.5	13.5
≥15	76.6	78.1	93.0	46.7	93.8	70.4	99.5	14.3
≥16	75.0	82.0	92.9	51.1	93.8	74.0	99.6	16.0
≥17	73.4	84.4	92.7	54.0	93.8	76.3	99.6	17.2
≥18	70.3	85.5	92.0	54.9	93.8	78.0	99.6	18.3
≥19	65.6	86.7	91.0	55.3	93.8	79.9	99.6	19.7
≥20	64.1	89.1	90.8	59.4	93.8	82.2	99.6	21.7
≥21	62.5	90.2	90.6	61.5	93.8	83.6	99.6	23.1
≥22	60.9	90.6	90.3	61.9	93.8	84.2	99.6	23.8
≥23	56.3	92.2	89.4	64.3	93.8	86.5	99.6	26.8
≥24	54.7	93.4	89.2	67.3	93.8	87.8	99.6	28.8
≥25	53.1	93.4	88.8	66.7	93.8	88.2	99.6	29.4
≥26	50.0	93.8	88.2	66.7	93.8	89.1	99.6	31.3
≥27	48.4	93.8	87.9	66.0	93.8	89.5	99.6	31.9
≥28	48.4	94.9	88.0	70.5	93.8	90.5	99.6	34.1
≥29	45.3	96.1	87.5	74.4	93.8	92.1	99.6	38.5
≥30	45.3	96.1	87.5	74.4	93.8	92.1	99.6	38.5
≥31	45.3	96.1	87.5	74.4	93.8	92.1	99.6	38.5
≥32	40.6	96.1	86.6	72.2	87.5	92.8	99.3	38.9
≥33	40.6	96.5	86.7	74.3	87.5	93.1	99.3	40.0
≥34	39.1	96.5	86.4	73.5	87.5	93.4	99.3	41.2
≥35	37.5	96.5	86.1	72.7	87.5	93.8	99.3	42.4
≥36	37.5	96.9	86.1	75.0	87.5	94.1	99.3	43.8
≥37	35.9	96.9	85.8	74.2	87.5	94.4	99.3	45.2
≥38	35.9	96.9	85.8	74.2	87.5	94.4	99.3	45.2
≥39	35.9	96.9	85.8	74.2	87.5	94.4	99.3	45.2
≥40	34.4	97.7	85.6	78.6	87.5	95.4	99.3	50.0
≥41	34.4	97.7	85.6	78.6	87.5	95.4	99.3	50.0
≥42	32.8	97.7	85.3	77.8	87.5	95.7	99.3	51.9

**≥43** [b]	**32.8**	**98.0**	**85.4**	**80.8**	87.5	96.1	99.3	53.8
≥44	32.8	98.0	85.4	80.8	87.5	96.1	99.3	53.8
≥45	32.8	98.0	85.4	80.8	87.5	96.1	99.3	53.8
≥46	32.8	98.4	85.4	84.0	87.5	96.4	99.3	56.0
≥47	29.7	98.4	84.8	82.6	87.5	97.0	99.3	60.9
≥48	29.7	98.4	84.8	82.6	87.5	97.0	99.3	60.9
≥49	29.7	98.8	84.9	86.4	87.5	97.4	99.3	63.6
≥50	29.7	98.8	84.9	86.4	87.5	97.4	99.3	63.6
≥51	29.7	98.8	84.9	86.4	87.5	97.4	99.3	63.6
≥52	29.7	98.8	84.9	86.4	87.5	97.4	99.3	63.6
≥53	29.7	98.8	84.9	86.4	87.5	97.4	99.3	63.6
≥54	29.7	98.8	84.9	86.4	87.5	97.4	99.3	63.6
≥55	28.1	98.8	84.6	85.7	87.5	97.7	99.3	66.7
≥56	28.1	98.8	84.6	85.7	87.5	97.7	99.3	66.7
≥57	28.1	98.8	84.6	85.7	87.5	97.7	99.3	66.7
≥58	28.1	99.2	84.7	90.0	87.5	98.0	99.3	70.0
≥59	26.6	99.2	84.4	89.5	81.3	98.0	99.0	68.4
≥60	25.0	99.2	84.1	88.9	75.0	98.0	98.7	66.7
≥61	25.0	99.2	84.1	88.9	75.0	98.0	98.7	66.7
≥62	25.0	99.2	84.1	88.9	75.0	98.0	98.7	66.7
≥63	25.0	99.2	84.1	88.9	75.0	98.0	98.7	66.7
≥64	25.0	99.2	84.1	88.9	75.0	98.0	98.7	66.7
≥65	25.0	99.2	84.1	88.9	75.0	98.0	98.7	66.7
≥66	25.0	99.2	84.1	88.9	75.0	98.0	98.7	66.7
≥67	25.0	99.2	84.1	88.9	75.0	98.0	98.7	66.7
≥68	25.0	99.2	84.1	88.9	75.0	98.0	98.7	66.7
≥69	25.0	99.2	84.1	88.9	75.0	98.0	98.7	66.7
≥70	25.0	99.6	84.2	94.1	75.0	98.4	98.7	70.6
≥71	23.4	99.6	83.9	93.8	75.0	98.7	98.7	75.0
≥72	20.3	99.6	83.3	92.9	62.5	98.7	98.0	71.4
≥73	18.8	99.6	83.1	92.3	56.3	98.7	97.7	69.2
≥74	17.2	99.6	82.8	91.7	50.0	98.7	97.4	66.7
≥75	15.6	99.6	82.5	90.9	43.8	98.7	97.1	63.6
≥76	15.6	99.6	82.5	90.9	43.8	98.7	97.1	63.6
≥77	15.6	99.6	82.5	90.9	43.8	98.7	97.1	63.6
≥78	14.1	99.6	82.3	90.0	43.8	99.0	97.1	70.0
≥79	12.5	100.0	82.1	100.0	37.5	99.3	96.8	75.0
≥80	12.5	100.0	82.1	100.0	37.5	99.3	96.8	75.0
≥81	12.5	100.0	82.1	100.0	37.5	99.3	96.8	75.0
≥82	9.4	100.0	81.5	100.0	37.5	100.0	96.8	100.0
≥83	9.4	100.0	81.5	100.0	37.5	100.0	96.8	100.0
≥84	6.3	100.0	81.0	100.0	25.0	100.0	96.2	100.0
≥85	6.3	100.0	81.0	100.0	25.0	100.0	96.2	100.0
≥86	6.3	100.0	81.0	100.0	25.0	100.0	96.2	100.0

≥87	3.1	100.0	80.5	100.0	12.5	100.0	95.6	100.0
≥88	3.1	100.0	80.5	100.0	12.5	100.0	95.6	100.0
≥89	3.1	100.0	80.5	100.0	12.5	100.0	95.6	100.0
≥90	3.1	100.0	80.5	100.0	12.5	100.0	95.6	100.0
≥91	3.1	100.0	80.5	100.0	12.5	100.0	95.6	100.0
≥92	1.6	100.0	80.3	100.0	6.3	100.0	95.3	100.0
≥93	1.6	100.0	80.3	100.0	6.3	100.0	95.3	100.0
≥94	1.6	100.0	80.3	100.0	6.3	100.0	95.3	100.0

IQ2+, the Informant Questionnaire for cognitive impairment–Two items (plus demographics); MCI, mild cognitive impairment; NPV, negative predictive value; PPV, positive predictive value.

[a] Prevalence of MCI/Dementia was readjusted to 20% in the Test sample, based on prior meta-analytic findings that community prevalence was ~15% for MCI and ~5% for dementia. In the Test sample, a subset of participants with MCI and dementia was randomly selected to readjust the prevalence in the dataset (see Methods section for further details). The resulting dataset comprised 256 participants with normal cognition (80%), 48 participants with MCI (15%), and 16 participants with dementia (5%).

[b] Bold-faced values indicate the two optimal cutoffs to detect MCI/dementia (i.e. the lower cutoff has high sensitivity and NPV, while the upper cutoff has high specificity and PPV).

Table S5. The test statistics of IQ2+ in identifying cognitive impairment in the Test sample, with the prevalence of MCI/Dementia artificially adjusted to 35%.[a]

Probability score (%)	MCI/Dementia versus Normal cognition				Dementia versus Non-dementia			
	Sensitivity (%)	Specificity (%)	NPV (%)	PPV (%)	Sensitivity (%)	Specificity (%)	NPV (%)	PPV (%)
≥4	100.0	4.8	100.0	36.1	100.0	3.5	100.0	10.3
≥5	98.2	21.2	95.7	40.1	100.0	16.0	100.0	11.7
≥6	92.9	40.4	91.3	45.6	100.0	31.9	100.0	14.0
≥7	89.3	54.8	90.5	51.5	100.0	43.8	100.0	16.5
≥8	89.3	63.5	91.7	56.8	100.0	50.0	100.0	18.2
≥9	87.5	72.1	91.5	62.8	100.0	56.9	100.0	20.5
≥10	83.9	73.1	89.4	62.7	100.0	59.0	100.0	21.3
**≥11** [b]	**82.1**	**73.1**	**88.4**	**62.2**	100.0	59.7	100.0	21.6
≥12	78.6	76.9	87.0	64.7	93.8	63.2	98.9	22.1
≥13	76.8	76.9	86.0	64.2	93.8	63.9	98.9	22.4
≥14	75.0	79.8	85.6	66.7	93.8	66.7	99.0	23.8
≥15	73.2	81.7	85.0	68.3	93.8	68.8	99.0	25.0
≥16	73.2	85.6	85.6	73.2	93.8	71.5	99.0	26.8
≥17	71.4	87.5	85.0	75.5	93.8	73.6	99.1	28.3
≥18	71.4	87.5	85.0	75.5	93.8	73.6	99.1	28.3
≥19	66.1	88.5	82.9	75.5	93.8	76.4	99.1	30.6
≥20	64.3	90.4	82.5	78.3	93.8	78.5	99.1	32.6
≥21	60.7	91.3	81.2	79.1	93.8	80.6	99.1	34.9
≥22	58.9	91.3	80.5	78.6	93.8	81.3	99.2	35.7
≥23	53.6	91.3	78.5	76.9	93.8	83.3	99.2	38.5
≥24	53.6	91.3	78.5	76.9	93.8	83.3	99.2	38.5
≥25	51.8	91.3	77.9	76.3	93.8	84.0	99.2	39.5
≥26	50.0	91.3	77.2	75.7	93.8	84.7	99.2	40.5
≥27	48.2	91.3	76.6	75.0	93.8	85.4	99.2	41.7
≥28	48.2	93.3	77.0	79.4	93.8	86.8	99.2	44.1
**≥29** [b]	**48.2**	**95.2**	**77.3**	**84.4**	93.8	88.2	99.2	46.9
≥30	48.2	95.2	77.3	84.4	93.8	88.2	99.2	46.9
≥31	48.2	95.2	77.3	84.4	93.8	88.2	99.2	46.9
≥32	42.9	96.2	75.8	85.7	87.5	90.3	98.5	50.0
≥33	42.9	96.2	75.8	85.7	87.5	90.3	98.5	50.0
≥34	42.9	96.2	75.8	85.7	87.5	90.3	98.5	50.0
≥35	41.1	96.2	75.2	85.2	87.5	91.0	98.5	51.9
≥36	41.1	96.2	75.2	85.2	87.5	91.0	98.5	51.9
≥37	39.3	96.2	74.6	84.6	87.5	91.7	98.5	53.8
≥38	39.3	96.2	74.6	84.6	87.5	91.7	98.5	53.8
≥39	39.3	96.2	74.6	84.6	87.5	91.7	98.5	53.8
≥40	37.5	97.1	74.3	87.5	87.5	93.1	98.5	58.3
≥41	37.5	97.1	74.3	87.5	87.5	93.1	98.5	58.3
≥42	37.5	97.1	74.3	87.5	87.5	93.1	98.5	58.3

≥43	37.5	97.1	74.3	87.5	87.5	93.1	98.5	58.3
≥44	37.5	97.1	74.3	87.5	87.5	93.1	98.5	58.3
≥45	37.5	97.1	74.3	87.5	87.5	93.1	98.5	58.3
≥46	37.5	97.1	74.3	87.5	87.5	93.1	98.5	58.3
≥47	33.9	97.1	73.2	86.4	87.5	94.4	98.6	63.6
≥48	33.9	97.1	73.2	86.4	87.5	94.4	98.6	63.6
≥49	33.9	97.1	73.2	86.4	87.5	94.4	98.6	63.6
≥50	33.9	97.1	73.2	86.4	87.5	94.4	98.6	63.6
≥51	33.9	97.1	73.2	86.4	87.5	94.4	98.6	63.6
≥52	33.9	98.1	73.4	90.5	87.5	95.1	98.6	66.7
≥53	33.9	98.1	73.4	90.5	87.5	95.1	98.6	66.7
≥54	33.9	98.1	73.4	90.5	87.5	95.1	98.6	66.7
≥55	33.9	98.1	73.4	90.5	87.5	95.1	98.6	66.7
≥56	33.9	98.1	73.4	90.5	87.5	95.1	98.6	66.7
≥57	33.9	98.1	73.4	90.5	87.5	95.1	98.6	66.7
≥58	33.9	98.1	73.4	90.5	87.5	95.1	98.6	66.7
≥59	32.1	98.1	72.9	90.0	81.3	95.1	97.9	65.0
≥60	30.4	98.1	72.3	89.5	75.0	95.1	97.2	63.2
≥61	30.4	98.1	72.3	89.5	75.0	95.1	97.2	63.2
≥62	30.4	98.1	72.3	89.5	75.0	95.1	97.2	63.2
≥63	30.4	98.1	72.3	89.5	75.0	95.1	97.2	63.2
≥64	30.4	98.1	72.3	89.5	75.0	95.1	97.2	63.2
≥65	30.4	98.1	72.3	89.5	75.0	95.1	97.2	63.2
≥66	30.4	98.1	72.3	89.5	75.0	95.1	97.2	63.2
≥67	30.4	98.1	72.3	89.5	75.0	95.1	97.2	63.2
≥68	30.4	98.1	72.3	89.5	75.0	95.1	97.2	63.2
≥69	30.4	98.1	72.3	89.5	75.0	95.1	97.2	63.2
≥70	30.4	98.1	72.3	89.5	75.0	95.1	97.2	63.2
≥71	28.6	98.1	71.8	88.9	75.0	95.8	97.2	66.7
≥72	25.0	98.1	70.8	87.5	62.5	95.8	95.8	62.5
≥73	23.2	98.1	70.3	86.7	56.3	95.8	95.2	60.0
≥74	21.4	98.1	69.9	85.7	50.0	95.8	94.5	57.1
≥75	17.9	98.1	68.9	83.3	43.8	96.5	93.9	58.3
≥76	17.9	98.1	68.9	83.3	43.8	96.5	93.9	58.3
≥77	17.9	98.1	68.9	83.3	43.8	96.5	93.9	58.3
≥78	17.9	98.1	68.9	83.3	43.8	96.5	93.9	58.3
≥79	16.1	99.0	68.7	90.0	37.5	97.2	93.3	60.0
≥80	16.1	99.0	68.7	90.0	37.5	97.2	93.3	60.0
≥81	16.1	99.0	68.7	90.0	37.5	97.2	93.3	60.0
≥82	12.5	99.0	67.8	87.5	37.5	98.6	93.4	75.0
≥83	12.5	99.0	67.8	87.5	37.5	98.6	93.4	75.0
≥84	8.9	99.0	66.9	83.3	25.0	98.6	92.2	66.7
≥85	8.9	99.0	66.9	83.3	25.0	98.6	92.2	66.7
≥86	8.9	99.0	66.9	83.3	25.0	98.6	92.2	66.7

≥87	5.4	99.0	66.0	75.0	12.5	98.6	91.0	50.0
≥88	5.4	100.0	66.2	100.0	12.5	99.3	91.1	66.7
≥89	5.4	100.0	66.2	100.0	12.5	99.3	91.1	66.7
≥90	5.4	100.0	66.2	100.0	12.5	99.3	91.1	66.7
≥91	5.4	100.0	66.2	100.0	12.5	99.3	91.1	66.7
≥92	3.6	100.0	65.8	100.0	6.3	99.3	90.5	50.0
≥93	3.6	100.0	65.8	100.0	6.3	99.3	90.5	50.0
≥94	3.6	100.0	65.8	100.0	6.3	99.3	90.5	50.0

IQ2+, the Informant Questionnaire for cognitive impairment–Two items (plus demographics); MCI, mild cognitive impairment; NPV, negative predictive value; PPV, positive predictive value.

[a] Prevalence of MCI/Dementia was readjusted to 35% in the Test sample, based on prior meta-lytic findings that community prevalence could be as high as ~25% for MCI and ~10% for dementia. In the Test sample, a subset of participants with MCI and dementia was randomly selected to readjust the prevalence in the dataset (see Methods section for further details). The resulting dataset comprised 104 participants with normal cognition (65%), 40 participants with MCI (25%), and 16 participants with dementia (10%).

[b] Bold-faced values indicate the two optimal cutoffs to detect MCI/dementia (i.e. the lower cutoff has high sensitivity and NPV, while the upper cutoff has high specificity and PPV).

Table S6. The test statistics of AD8 in identifying cognitive impairment in the Test sample.

Score	MCI/Dementia versus Normal cognition				Dementia versus Non-dementia			
	Sensitivity (%)	Specificity (%)	NPV (%)	PPV (%)	Sensitivity (%)	Specificity (%)	NPV (%)	PPV (%)
≥1	72.4	64.9	93.7	24.6	100.0	61.6	100.0	7.1
≥2	60.5	84.6	93.1	38.3	100.0	80.8	100.0	13.3
≥3	48.7	92.3	91.9	50.0	100.0	89.3	100.0	21.6
≥4	42.1	94.4	91.2	54.2	100.0	92.1	100.0	27.1
≥5	35.5	98.1	90.6	75.0	100.0	96.3	100.0	44.4
≥6	27.6	99.2	89.7	84.0	93.8	98.2	99.8	60.0
≥7	19.7	100.0	88.7	100.0	75.0	99.4	99.3	80.0
≥8	13.2	100.0	87.9	100.0	50.0	99.6	98.5	80.0

AD8, the Eight-item Informant Interview to Differentiate Aging and Dementia; MCI, mild cognitive impairment; NPV, negative predictive value; PPV, positive predictive value.

Table S7. The test statistics of AD8 in identifying cognitive impairment in the Test sample, with the prevalence of MCI/Dementia artificially adjusted to 20%.[a]

Score	MCI/Dementia versus Normal cognition				Dementia versus Non-dementia			
	Sensitivity (%)	Specificity (%)	NPV (%)	PPV (%)	Sensitivity (%)	Specificity (%)	NPV (%)	PPV (%)
≥1	73.4	66.4	90.9	35.3	100.0	61.5	100.0	12.0
≥2	60.9	85.2	89.7	50.6	100.0	79.9	100.0	20.8
≥3	50.0	92.2	88.1	61.5	100.0	88.2	100.0	30.8
≥4	43.8	94.1	87.0	65.1	100.0	91.1	100.0	37.2
≥5	37.5	97.7	86.2	80.0	100.0	95.4	100.0	53.3
≥6	31.3	98.8	85.2	87.0	93.8	97.4	99.7	65.2
≥7	21.9	100.0	83.7	100.0	75.0	99.3	98.7	85.7
≥8	15.6	100.0	82.6	100.0	50.0	99.3	97.4	80.0

AD8, the Eight-item Informant Interview to Differentiate Aging and Dementia; MCI, mild cognitive impairment; NPV, negative predictive value; PPV, positive predictive value.

[a] Prevalence of MCI/Dementia was readjusted to 20% in the Test sample, based on prior meta-analytic findings that community prevalence was ~15% for MCI and ~5% for dementia. In the Test sample, a subset of participants with MCI and dementia was randomly selected to readjust the prevalence in the dataset (see Methods section for further details). The resulting dataset comprised 256 participants with normal cognition (80%), 48 participants with MCI (15%), and 16 participants with dementia (5%).

Table S8. The test statistics of AD8 in identifying cognitive impairment in the Test sample, with the prevalence of MCI/Dementia artificially adjusted to 35%.[a]

Score	MCI/Dementia versus Normal cognition				Dementia versus Non-dementia			
	Sensitivity (%)	Specificity (%)	NPV (%)	PPV (%)	Sensitivity (%)	Specificity (%)	NPV (%)	PPV (%)
≥1	69.6	59.6	78.5	48.1	100.0	54.9	100.0	19.8
≥2	58.9	81.7	78.7	63.5	100.0	75.0	100.0	30.8
≥3	50.0	88.5	76.7	70.0	100.0	83.3	100.0	40.0
≥4	44.6	94.2	76.0	80.6	100.0	89.6	100.0	51.6
≥5	39.3	98.1	75.0	91.7	100.0	94.4	100.0	66.7
≥6	33.9	99.0	73.6	95.0	93.8	96.5	99.3	75.0
≥7	25.0	100.0	71.2	100.0	75.0	98.6	97.3	85.7
≥8	16.1	100.0	68.9	100.0	50.0	99.3	94.7	88.9

AD8, the Eight-item Informant Interview to Differentiate Aging and Dementia; MCI, mild cognitive impairment; NPV, negative predictive value; PPV, positive predictive value.

[a] Prevalence of MCI/Dementia was readjusted to 35% in the Test sample, based on prior meta-lytic findings that community prevalence could be as high as ~25% for MCI and ~10% for dementia. In the Test sample, a subset of participants with MCI and dementia was randomly selected to readjust the prevalence in the dataset (see Methods section for further details). The resulting dataset comprised 104 participants with normal cognition (65%), 40 participants with MCI (25%), and 16 participants with dementia (10%).

ADDITIONAL REFERENCES

1. Tomaszewski Farias S, Mungas D, Harvey DJ, Simmons A, Reed BR, Decarli C. The measurement of everyday cognition: development and validation of a short form of the Everyday Cognition scales. *Alzheimers Dement.* 2011;7(6):593-601.
2. Ng TP, Niti M, Chiam PC, Kua EH. Physical and cognitive domains of the Instrumental Activities of Daily Living: validation in a multiethnic population of Asian older adults. *J Gerontol A Biol Sci Med Sci.* 2006;61(7):726-735.
3. Katz S, Downs TD, Cash HR, Grotz RC. Progress in development of the index of ADL. *Gerontologist.* 1970;10(1):20-30.
4. Kaufer DI, Cummings JL, Ketchel P, et al. Validation of the NPI-Q, a brief clinical form of the Neuropsychiatric Inventory. *J Neuropsychiatry Clin Neurosci.* 2000;12(2):233-239.
5. Kroenke K, Spitzer RL, Williams JB. The PHQ-9: validity of a brief depression severity measure. *J Gen Intern Med.* 2001;16(9):606-613.
6. Spitzer RL, Kroenke K, Williams JB, Löwe B. A brief measure for assessing generalized anxiety disorder: the GAD-7. *Arch Intern Med.* 2006;166(10):1092-1097.
7. Nasreddine ZS, Phillips NA, Bedirian V, et al. The Montreal Cognitive Assessment, MoCA: a brief screening tool for mild cognitive impairment. *J Am Geriatr Soc.* 2005;53(4):695-699.
8. Ang LC, Yap P, Tay SY, Koay WI, Liew TM. Examining the Validity and Utility of Montreal Cognitive Assessment Domain Scores for Early Neurocognitive Disorders. *J Am Med Dir Assoc.* 2023;24(3):314-320 e312.
9. Weintraub S, Besser L, Dodge HH, et al. Version 3 of the Alzheimer Disease Centers' Neuropsychological Test Battery in the Uniform Data Set (UDS). *Alzheimer Dis Assoc Disord.* 2018;32(1):10-17.
10. Inouye SK. *The Short Confusion Assessment Method (Short CAM): Training Manual and Coding Guide.* Boston: Hospital Elder Life Program; 2014.
11. van Harten AC, Mielke MM, Swenson-Dravis DM, et al. Subjective cognitive decline and risk of MCI: The Mayo Clinic Study of Aging. *Neurology.* 2018;91(4):e300-e312.
12. Canevelli M, Adali N, Voisin T, et al. Behavioral and psychological subsyndromes in Alzheimer's disease using the Neuropsychiatric Inventory. *Int J Geriatr Psychiatry.* 2013;28(8):795-803.
13. Liew TM. Neuropsychiatric symptoms in cognitively normal older persons, and the association with Alzheimer's and non-Alzheimer's dementia. *Alzheimers Res Ther.* 2020;12(1):35.
14. Yatawara C, Hiu S, Tan L, Kandiah N. Neuropsychiatric symptoms in South-East Asian patients with mild cognitive impairment and dementia: prevalence, subtypes, and risk factors. *International journal of geriatric psychiatry.* 2018;33(1):122-130.
15. Kroenke K, Wu J, Yu Z, et al. Patient Health Questionnaire Anxiety and Depression Scale: Initial Validation in Three Clinical Trials. *Psychosom Med.* 2016;78(6):716-727.
16. Kroenke K, Spitzer RL, Williams JB, Monahan PO, Löwe B. Anxiety disorders in primary care: prevalence, impairment, comorbidity, and detection. *Ann Intern Med.* 2007;146(5):317-325.
17. Kroenke K, Spitzer RL, Williams JB, Löwe B. The Patient Health Questionnaire Somatic, Anxiety, and Depressive Symptom Scales: a systematic review. *Gen Hosp Psychiatry.* 2010;32(4):345-359.
18. Tsoi KF, Chan JC, Hirai HW, Wong SS, Kwok TY. Cognitive tests to detect dementia: A systematic review and meta-analysis. *JAMA Internal Medicine.* 2015;175(9):1450-1458.
19. Breton A, Casey D, Arnaoutoglou NA. Cognitive tests for the detection of mild cognitive impairment (MCI), the prodromal stage of dementia: Meta-analysis of diagnostic accuracy studies. *Int J Geriatr Psychiatry.* 2019;34(2):233-242.

20. Liew TM, Feng L, Gao Q, Ng TP, Yap P. Diagnostic utility of Montreal Cognitive Assessment in the Fifth Edition of Diagnostic and Statistical Manual of Mental Disorders: major and mild neurocognitive disorders. *J Am Med Dir Assoc.* 2015;16(2):144-148.
21. Craft S, Newcomer J, Kanne S, et al. Memory improvement following induced hyperinsulinemia in Alzheimer's disease. *Neurobiol Aging.* 1996;17(1):123-130.
22. Possin KL, Laluz VR, Alcantar OZ, Miller BL, Kramer JH. Distinct neuroanatomical substrates and cognitive mechanisms of figure copy performance in Alzheimer's disease and behavioral variant frontotemporal dementia. *Neuropsychologia.* 2011;49(1):43-48.
23. Morris JC. The Clinical Dementia Rating (CDR): current version and scoring rules. *Neurology.* 1993;43(11):2412-2414.
24. Rikkert MG, Tona KD, Janssen L, et al. Validity, reliability, and feasibility of clinical staging scales in dementia: a systematic review. *Am J Alzheimers Dis Other Demen.* 2011;26(5):357-365.
25. Hanley JA, McNeil BJ. A method of comparing the areas under receiver operating characteristic curves derived from the same cases. *Radiology.* 1983;148(3):839-843.
26. Hastie T, Tibshirani R, Friedman J. *The elements of statistical learning: data mining, inference, and prediction.* New York: Springer-Verlag; 2009.
